# Dislocation Density‐Mediated Functionality in Single‐Crystal BaTiO_3_

**DOI:** 10.1002/advs.202403550

**Published:** 2024-06-17

**Authors:** Fangping Zhuo, Xiandong Zhou, Felix Dietrich, Mehrzad Soleimany, Patrick Breckner, Pedro B. Groszewicz, Bai‐Xiang Xu, Gerd Buntkowsky, Jürgen Rödel

**Affiliations:** ^1^ Department of Materials and Earth Sciences Technical University of Darmstadt 64287 Darmstadt Germany; ^2^ Failure Mechanics and Engineering Disaster Prevention Key Laboratory of Sichuan Province College of Architecture and Environment MOE Key Laboratory of Deep Earth Science and Engineering College of Architecture and Environment Sichuan University Chengdu 610065 China; ^3^ Institute of Physical Chemistry Technical University of Darmstadt 64287 Darmstadt Germany; ^4^ Department of Radiation Science and Technology Delft University of Technology Delft 2629JB Netherlands

**Keywords:** dislocations, ferroelectrics, functional ceramics, plastic deformation

## Abstract

Unlike metals where dislocations carry strain singularity but no charge, dislocations in oxide ceramics are characterized by both a strain field and a local charge with a compensating charge envelope. Oxide ceramics with their deliberate engineering and manipulation are pivotal in numerous modern technologies such as semiconductors, superconductors, solar cells, and ferroics. Dislocations facilitate plastic deformation in metals and lead to a monotonous increase in the strength of metallic materials in accordance with the widely recognized Taylor hardening law. However, achieving the objective of tailoring the functionality of oxide ceramics by dislocation density still remains elusive. Here a strategy to imprint dislocations with {100}<100> slip systems and a tenfold change in dislocation density of BaTiO_3_ single crystals using high‐temperature uniaxial compression are reported. Through a dislocation density‐based approach, dielectric permittivity, converse piezoelectric coefficient, and alternating current conductivity are tailored, exhibiting a peak at medium dislocation density. Combined with phase‐field simulations and domain wall potential energy analyses, the dislocation‐density‐based design in bulk ferroelectrics is mechanistically rationalized. These findings may provide a new dimension for employing plastic strain engineering to tune the electrical properties of ferroics, potentially paving the way for advancing dislocation technology in functional ceramics.

## Graphical abstract

A novel strategy for engineering dielectric permittivity, converse piezoelectric coefficient, and alternating current conductivity through a dislocation density‐based approach is introduced. This innovative dislocation‐centric design within bulk ferroelectrics represents a concept with the potential for broad application to other materials, potentially ushering in a new era for dislocation.

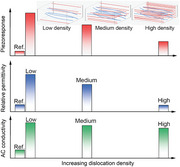

## 1 Introduction

Dislocations, as 1D topological crystalline defects, are often perceived as having a detrimental effect on the functional properties of classic semiconductors.^[^ ^1^, ^2^ ^]^ In dislocation‐mediated plasticity, they serve as the fundamental building blocks that play a pivotal role in governing the plastic deformation and damage evolution across various materials, including semi‐crystalline polymers,^[^ ^3^ ^]^ semiconductors,^[^ ^4^, ^5^, ^6^ ^]^ metallic materials,^[^ ^1^, ^7^ ^]^ and composites.^[^ ^8^ ^]^ It is widely acknowledged that the strength of bulk metallic materials monotonically increases with an increase in dislocation density (Figure S1, Supporting Information), following the well‐established Taylor strengthening law.^[^ ^9^, ^10^ ^]^ However, excessively high dislocation density can lead to a decline in strength, known as dislocation exhaustion or softening.^[^ ^11^ ^]^ This well‐known relationship highlights the significant influence of dislocation density on enhancing the strength and mechanical properties of metals and alloys, underscoring their importance in understanding and manipulating material behavior. In contrast to metals, where dislocations display only a strain singularity, dislocations in ceramics can feature both strain and charge.^[^ ^12^, ^13^ ^]^ Although the slip systems are limited to ceramic materials, the introduced dislocations are commonly observed to be nearly straight and parallel to each other.^[^ ^12^, ^14^, ^15^, ^16^, ^17^ ^]^ This characteristic alignment of 1D dislocations is a notable feature in structural and functional ceramics. It arises from the specific crystal structure and bonding arrangements within ceramics, which favor the creation of such linear and parallel dislocation configurations. Over the last five years, charged dislocations in ceramics have attracted growing attention due to their capacity to modulate composition, strain, and charge over broad length scales, thus offering an extra degree of freedom for finely tailoring electrical, optical and thermal properties beyond limits inherent in bulk doping.^[^ ^15^, ^16^, ^17^, ^18^, ^19^ ^]^ Despite such excitement, the deliberate creation and control of dislocations (e.g., arrangement and density) in ceramics, particularly through mechanical manufacturing methods, remain significant challenges that limit their full potential.

Ferroelectrics, classified as a sub‐class of piezoelectric materials, possess a polar structure that can be switched by the application of an electric field. They have garnered wide‐ranging applications in modern electronics and memory systems,^[^ ^20^, ^21^, ^22^, ^23^, ^24^ ^]^ such as sensors, actuators, transducers, and ferroelectric random‐access memory. In bulk ferroelectrics, achieving high intrinsic electromechanical response and effectively controlling the mobility of ferroelectric domain walls are significant challenges.^[^ ^25^, ^26^ ^]^ Current strategies to enhance ferroelectric properties encompass a range of state‐of‐the‐art concepts, including strain engineering,^[^ ^27^ ^]^ point defect engineering,^[^ ^28^ ^]^ polarization rotation,^[^ ^29^ ^]^ and phase boundary engineering.^[^ ^23^, ^30^, ^31^ ^]^ Furthermore, common approaches to regulate the motion of domain walls include domain‐wall engineering,^[^ ^32^, ^33^ ^]^ point defect doping,^[^ ^34^ ^]^ and the incorporation of precipitates or secondary phase particles.^[^ ^35^, ^36^ ^]^ The ordering of chemical defects in ferroelectrics has been suggested to occur through short‐range migration of point defects.^[^ ^34^ ^]^ However, this process requires a specific amount of time (e.g., aging) or thermal treatment, such as quenching, to reach completion. Despite great efforts, achieving controlled and ordered distributions of point defects and volumetric defects remains a grand challenge.

Owing to their intrinsic strong interplay between lattice (or strain) and charge (or polarization) degrees of freedom, introducing 1D dislocation defects into ferroelectrics yields three primary effects: First, dislocations act as nucleation sites, facilitating the formation of ferroelectric domains.^[^ ^37^, ^38^ ^]^ Second, they serve as pinning centers that impede the motion of domain walls,^[^ ^26^, ^39^, ^40^ ^]^ which are known as 2D topological interfacial defects.^[^ ^32^ ^]^ Lastly, dislocations mediate domain switching and significantly influence the overall stability of the ferroelectric domains.^[^ ^41^ ^]^ The utilization of misfit dislocations has unveiled tremendous potential for unprecedented properties of epitaxial ferroelectric thin films.^[^ ^42^, ^43^ ^]^ For example, strained BaTiO_3_ thin films demonstrated a remarkable improvement of 250% in remanent polarization,^[^ ^43^ ^]^ while room‐temperature ferroelectricity was achieved in paraelectric SrTiO_3_ thin films^.[^ ^42^ ^]^ However, interfacial dislocations are often a nuisance in thin‐film ferroelectrics and are held responsible for the degradation of ferroelectric films,^[^ ^44^, ^45^, ^46^ ^]^ due to the formation of a dead layer or relaxation of near‐surface eigenstrain. Fortunately, there is a natural way to circumvent the occurrence of these dislocation‐related detrimental aspects in thin‐film ferroelectrics, by permanently imprinting dislocations into the bulk of the material rather than at the interface. For instance, we recently demonstrated that a mechanically introduced dislocation network (“1D self‐doping”) allows access to a 19‐fold increase in electromechanical response in deformed bulk single‐crystal BaTiO_3_ with {101}<101> slip systems when loaded along the [001] direction.^[^ ^26^ ^]^ The interactions between dislocations and domain walls not only provided a macroscopic restoring force but also enabled local domain wall pinning for dielectric and piezoelectric response. Furthermore, direct control over anisotropic electromechanical properties and stability by leveraging the dislocation‐domain wall interactions was successfully achieved, capitalizing on the anisotropy of domain wall pinning forces.^[^ ^41^, ^47^, ^48^ ^]^ To tune the functional properties of ferroelectric ceramic oxides, a necessary prerequisite is to engineer dislocations with controllable density. While this understanding is well‐established in metals, but remains elusive in our previous reports,^[^ ^26^, ^41^ ^]^ where the imprinted dislocation density is estimated to be up to ≈1012 m^−2^ and is still poorly understood in ferroic oxides. Ideally, the ability to control the spatial alignment of dislocations, as well as their mesostructure and interaction with domain walls, is also desirable. Nevertheless, the influence of dislocation density on the physical properties of ferroelectrics remains largely unexplored because the inherent brittleness of ferroelectric oxides, arising from their strong and covalent bonding, poses a significant obstacle to furnishing ferroelectrics with controlled dislocation density.^[^ ^49^, ^50^ ^]^ As a result, disclosing the effects of dislocation density and developing strategies for precisely managing it in bulk ferroelectrics hold the potential for overcoming the limitations and unlocking new opportunities in ferroics.

In the present work, we revisit a model system of single‐crystal BaTiO_3_, due to its well‐known ferroelectric properties and the presence of 180° ferroeletric domain walls and 90° ferroelastic domain walls which can be affected by the dislocation‐associated lattice strains. Introducing dislocations with controlled density into BaTiO_3_ single crystals is accessible by compressive plastic deformation at high temperatures, without and with a notch to focus the strain concentration. Contrary to the widely recognized Taylor hardening law, which suggests that the strength of metallic materials reaches a minimum point, we demonstrated that dielectric and piezoelectric responses, along with alternating current (AC) conductivity, attain their peak values at specific dislocation density. Phase‐field simulations were successfully utilized to rationalize the linear relationship between dislocation density and local domain‐wall pinning field and the significant impact of dislocation density on the macroscopic ferroelectric hysteresis loops and electrical properties.

## 2 Results and Discussion

### 2.1 Introducing Dislocations with Different Density by High‐Temperature Compression

**Figure 1:**
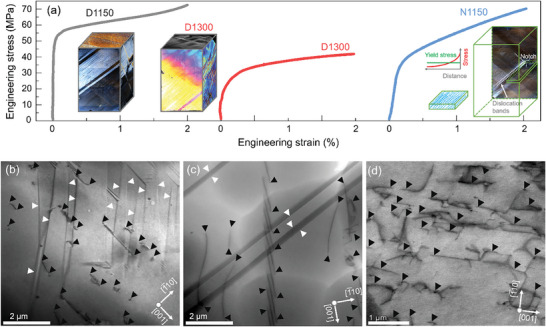
a) Stress–strain curves of samples deformed at 1150 °C (D1150), deformed at 1300 °C (D1300) and deformed at 1150 °C with a notch (N1150). The insets provide the optical images taken by a laser scanning microscope in the reflection mode after the deformation. Representative bright‐field transmission electron microscopy (BF‐TEM) images of b) D1150, c) D1300 and d) N1150. All bright‐field TEM images were viewed on the (110) plane. The positions of dislocations and domain walls are depicted by black and white arrowheads, respectively.

Uniaxial compression of [110]‐oriented BaTiO_3_ single crystals at 1150 °C (hereafter named D1150) and 1300 °C (hereafter named D1300) generated mesoscopic dislocation structures (**Figure** **1**a and the Experimental Section for details). After deformation, we extracted slices for the observation of dislocation structures both parallel and perpendicular to the deformation direction [110] (see schematics in Figure S2, Supporting Information). D1150 samples have a dislocation density of ≈1.8 × 1012 m^−2^, corresponding to an average dislocation spacing of 2 µm, see electron channeling contrast imaging (ECCI) image (Figure S3a, Supporting Information) and transmission electron microscopy (TEM) images (Figure S4, Supporting Information). The dislocation density of the D1300 samples is ≈7.0 × 1012 m^−2^, which corresponds to an average spacing between two dislocations of ≈540 nm (Figure S3b; Figure S5, Supporting Information). The high‐temperature uniaxial compression resulted in a homogenous distribution of dislocations, where extended dislocations with the {100}<100> slip systems run along the [001] direction in a parallel fashion (see TEM images in Figure 1b,c). Elaborating on the atomic structure of a BaTiO_3_ dislocation necessitates advanced transmission electron microscopy to visualize the polarization and charge distribution surrounding the dislocation cores.^[^ ^51^, ^52^ ^]^ It was reported that the dislocation density of reference undeformed crystals is 108–109 m^−2^,^[^ ^53^, ^54^ ^]^ indicating an average distance between two dislocations of ≈20 µm.

To achieve a yet higher dislocation density, a notched sample was deformed at 1150 °C (hereafter named N1150) using a stress concentration (Figure 1a and the Experimental Section). This advanced technique has been employed to introduce dense and well‐oriented dislocations in SrTiO_3_ single crystals before, where the dislocation density was increased by one order of magnitude up to 1013 m^−2^.^[^ ^18^ ^]^ {100}<100> is the slip system of BaTiO_3_ that can be activated at elevated temperature,^[^ ^41^, ^53^ ^]^ which is evident at one of the {100} sides (slip bands are marked by white dashed lines in Figure 1a) at a 45° angle, similar to the results for SrTiO_3_.^[^ ^15^, ^55^ ^]^ All stress–strain curves were plotted in Figure 1a, and the clear plastic deformation regime provided a unique opportunity to align dislocations within the sample along their slip planes. The presence of a notch (Figure 1a) resulted in a localized stress concentration, leading to the formation of dislocation bands that are distributed in an inhomogeneous manner. At first sight, it appears as if D1150 features considerable strain hardening, however, this merely reflects the inhomogeneous stress distribution around the notch with enhanced strain requiring dislocations moving into locally lower stress fields. To accurately quantify the dislocation density, we acquired TEM and ECCI images from various regions of the deformed samples. The deformed sample with a notch exhibited the highest dislocation density, measuring ≈1.5 × 1013 m^−2^, corresponding to an average spacing of ≈250 nm (Figure S6, Supporting Information).

In ferroelectrics, dislocations, and their associated local tensile and compressive stress fields act as sites for the formation of domains. The strain fields accompanying the dislocation lines introduce crystalline anisotropy and favor electrical dipoles with orientation perpendicular to the dislocation lines. This prompts the preferred formation of domains with polarization vectors perpendicular to the [001] direction.^[^ ^41^ ^]^ In contrast, the reference undeformed sample naturally prompted *a*_1_/*c*, *a*_2_/*c*, and *a*_1_/*a*_2_ 90° domain walls (see optical images in Figure S7, Supporting Information), which were formed during the cooling process from above the Curie temperature. Note that the *a*_1_ and *a*_2_ domains have polarization vectors aligned parallel to the [100] and [010] directions, respectively, while the *c* domains have polarization vectors parallel to the [001] direction. We find clear evidence of deformation‐controlled domain nucleation and macroscopic effects of dislocations on domain patterns in the deformed samples (Figure 1a). As expected, our directed dislocation imprint resulted in the nucleation of *a*_1_ and *a*_2_ domains (see optical images in Figures S8–S10, Supporting Information). More precisely, the spontaneous polarization vectors of individual domains have the propensity to align along either the [100] or the [010] in‐plane tetragonal directions, owing to the planar strain field surrounding the dislocations. Rather unexpectedly, the mechanical imprint is macroscopically reflected in the curvature and the size of 90° domain walls. The transition from *a*/*c* to *a*_1_/*a*_2_ domain structure was further validated by piezoresponse force microscopy (PFM) and X‐ray diffraction (XRD) results, as displayed in Figures S11 and S12, Supporting Information. The average domain size increases from 8 µm to 65 µm with the dislocation density increasing from 1.1 × 1012 to 1.5 × 1013 m^−2^. This yields a platform for revealing the electrical properties influenced by dislocation density,^[^ ^54^ ^]^ both in the small and large signal regimes.

### 2.2 Influence of Dislocation Density on Electrical Properties

Having established the correlation between mesoscopic domain structure and dislocation density‐related strain, we now pose fundamental questions regarding the extent to which dislocations can pin domain walls and their potential contributions to electrical properties. In fact, BaTiO_3_ single crystals are an ideal platform to explore dislocation density‐tuned functionality in ferroelectrics because the dislocation‐associated strain fields are best exposed in a tetragonal phase with 180° ferroelectric and 90° ferroelastic domain walls. When dislocations are introduced into BaTiO_3_ and become embedded within these domain walls, they will, in turn, have a significant influence on dielectric, ferroelectric, and electromechanical properties. Aiming to study the macroscopic effects of the dislocations, we first examined the small‐signal (1 V, root‐mean‐square) dielectric permittivity (*ε*_33_) as a function of temperature up to 200 °C (Figure S13, Supporting Information). The Curie point of the deformed samples with different dislocation densities was slightly enhanced as compared to that of the reference sample, which is due to the dislocation‐induced strain fields.^[^ ^41^ ^]^ Notably, the dielectric permittivity of the deformed samples with *a*‐domains increased as compared to the reference sample with both *a*‐ and *c*‐domains due to the anisotropic dielectric tensor of BaTiO_3_, where *ε*_a_ > *ε*_c_.^[^ ^56^ ^]^ Owing to the varying extrinsic contributions from the domain walls to permittivity, a clear picture of the influence of dislocation density on the small‐signal dielectric response has not yet been drawn.

**Figure 2:**
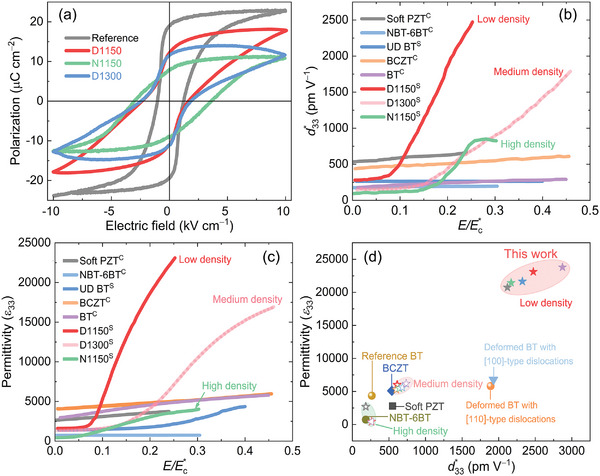
a) Room temperature polarization versus electric field hysteresis loops of reference and deformed samples (D1150, N1150, and D1300) quantified at a frequency of 1 Hz. b) Converse piezoelectric coefficient (*d*_33_*) and c) corresponding dielectric permittivity (*ε*_33_) as a function of AC field (normalized by coercive field *E*_c_*) for various samples measured at 1 kHz. Soft PZT: modified lead zirconate titanate (PIC 151); UD BT: undeformed BaTiO_3_; BT: BaTiO_3_; BCZT: Ba_0.85_Ca_0.15_Zr_0.1_Ti_0.9_O_3_; NBT‐6BT: 0.94(Na_1/2_Bi_1/2_)TiO_3_–0.06BaTiO_3_. C indicates polycrystalline ceramics while S stands for single crystals. The *E*_c_* is defined as (|*E*_c_^+^| + |*E*_c_^−^|)/2, where |*E*_c_^+^| and |*E*_c_^−^| are the absolute values of the positive and negative coercive fields, respectively. The relationship between *ε*_33_ and *d*_33_* at a field of 50 V mm^−1^ for different materials was plotted in (d).

The application of large‐signal super‐coercive fields emphasizes the substantial influence of dislocation density on room‐temperature polarization hysteresis loops, as depicted in **Figure** **2**a. Macroscopically, an increase in dislocation density resulted in a decrease in maximum polarization (*P*_max_) and remanent polarization (*P*_r_) and a proportional increase in apparent coercive electric field (*E*_c_*). Indeed, the imprinted dislocations can act as pinning sites for the motion of domain walls in single‐crystal BaTiO_3_, leading to an increase of *E*_c_*, as extensively investigated in ferroelectrics. By examining the shape of these polarization loops observed in both deformed and undeformed samples (Figure 2a), it is evident that the presence of dislocation networks is responsible for the asymmetric deformation of the polarization loops. This effect is similar to the impact caused by other defects, such as oxygen vacancies and precipitates.^[^ ^34^, ^35^ ^]^

We activated the high‐temperature slip system of {100}<100>, which has six different glide planes, (Figure S14b, Supporting Information). There are two categories of interrelationships between activated slip planes and domain wall variants. When slip planes have a 45° angle with respect to the deformation direction [110], *a*_1_‐*a*_2_ 90° domain walls could be parallel to dislocation lines, ensuring the line‐plane interactions between dislocations and domain walls. Our solid‐state Nuclear Magnetic Resonance (NMR) spectroscopy demonstrated that the poled sample had only *a*_1_‐ and *a*_2_‐domains even if the sample was rotated from 0° to 50° (see angle‐dependent NMR data in Figure S14, Supporting Information).

According to previous reports,^[^ ^26^, ^41^, ^47^, ^48^ ^]^ the full potential of the interactions between 1D dislocations and 2D domain walls resides in the sub‐coercive field regime (below 0.5*E*_c_*). We simultaneously quantified the sub‐coercive field permittivity, *ε*_33_, and converse piezoelectric coefficient, *d*_33_*, see details in the Experimental Section. As featured in Figure 2b,c, the large‐signal *d*_33_* and *ε*_33_ of D1150 and D1300 (also N1150) increased exponentially at *E*/*E*_c_* = 0.08 and at *E*/*E*_c_* = 0.15, respectively. In the case of the D1150 sample, which exhibited a low dislocation density of 1.1 × 1012 m^−2^, we observed an ≈8‐fold increase in *d*_33_* and a 9‐fold increase in *ε*_33_ as compared to the undeformed reference (UD BT) sample (*d*_33_* ≈263 pm V^−1^, *ε*_33_ ≈ 2204 at *E*/*E*_c_* = 0.25). This is because the contribution of 180° domain walls to the piezoelectric coefficient is considered to be negligible, but is recognized to affect the dielectric response. The significant subcoercive *ε*_33_ demonstrates a notable frequency dependency (Figure S15, Supporting Information), suggesting dynamic interactions between dislocations and domain walls. It is worth noting that we observed inverse domain‐size dependence of dielectric response in the unpoled deformed samples (see temperature‐dependent *ε*_33_ data below 100 °C in Figure S13, Supporting Information), which can be understood in light of the corresponding change in the propensity of polarization rotation inside the domains.^[^ ^57^ ^]^ This suggests that variations in domain size or domain wall density have a minimal impact on the dielectric and piezoelectric responses. The pinning field increased from 14 V mm^−1^ to 57 V mm^−1^ with increasing dislocation density from 1.1 × 1012 to 1.5 × 1013 m^−2^ (see Table 1). As compared to other material systems, in our case of mechanical imprint, the density is only changed by a factor of ten or so, and the relevant effects are fully captured. The AC field applied to all materials discussed herein is maintained below 0.5*E*_c_* to effectively mitigate any contributions from domain switching to both dielectric and piezoelectric responses. When we kept the AC field at 50 V mm^−1^ and plotted the *d*_33_* versus *ε*_33_, the potential of the dislocation density‐tuned dislocation‐domain wall interaction was obtained (Figure 2d). Polarization hysteresis loops for various ceramics are plotted in Figure S16, (Supporting Information). For the notched sample with a high density of ≈1.5 × 1013 m^−2^, the mobility and density of domain walls can be largely suppressed, leading to reduced piezoelectric and dielectric properties. The interaction between domain walls and dislocations may reach saturation and subsequently begin to decrease (see the data for the N1150 sample in Figure 2b), which could be associated with domain switching. Our reported *d*_33_* of 2473 pm V^−1^ and *ε*_33_ values of *ε*_33_ ≈ 23 100 at *E*/*E*_c_* = 0.25 are higher than those of the sample with the {110}<110> slip systems (the [110]‐type dislocations). Interestingly, the inverse values of both *d*_33_* and dielectric permittivity display distinct minimum values (Figure S17c, Supporting Information), evoking similarities to the Taylor hardening law (Figure S1, Supporting Information). Note that the dislocation density‐tuned dielectric permittivity and piezoelectric coefficient values remained highly reproducible (Figure S18, Supporting Information and ref ^[^ ^26^ ^]^).

**Table 1:** Comparison of small‐signal and large‐signal properties of reference and deformed BaTiO_3_ samples.

Sample	*ρ*_D_ [m^−2^]	*P*_max_ [µC cm^−2^]	*P*_r_ [µC cm^−2^]	*E*_c_ ^a)^ [kV cm^−1^]	*E*_pin_ [V mm^−1^]	*d*_33_^*^ [pm V^−1^]	*ε*_33_/*ε*_0_
Reference	108–109	22.9	19.1	1.02	0	263	4350
D1150	1.8 × 1012	17.7	12.6	1.98	14	2473	23 100
D1300	7.0 × 10^12^	13.9	11.5	2.17	29	1788	16 930
N1150	1.5 × 10^13^	10.8	7.9	3.31	57	825	4030

^a)^ *E*_c_ is defined as (|*E*_c_^+^| + |*E*_c_^–^|)/2, where |*E*_c_^+^| and |*E*_c_^–^| are the absolute values of the positive and negative coercive fields, respectively.

**Figure 3:**
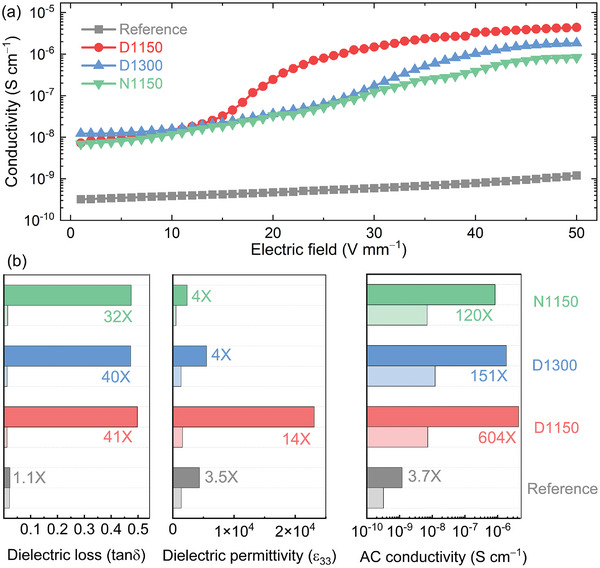
a) AC conductivity as a function of electric field for the reference and deformed samples (D1150, N1150, and D1300). b) Changes of dielectric permittivity, loss, and AC conductivity under AC field of 1 and 50 V mm^−1^.

The introduction of charged dislocations through plastic deformation has demonstrated the ability to enhance the electrical conductivity of oxide ceramics.^[^ ^12^, ^15^, ^16^, ^58^ ^]^ This modification of electrical properties carries great importance in various energy applications, including photoconductivity, photovoltaics, and electrochemistry.^[^ ^18^, ^59^, ^60^ ^]^ In general, the AC conductivity is given by^[^ ^43^ ^]^

(1) $$\sigma_{ac}\;=\;2\pi \omega \varepsilon_{r}\varepsilon_{0}\tan\delta$$

where ω is the frequency, *ε*_r_, and *ε*_0_ are the relative permittivity and vacuum permittivity, tan*δ* is the dielectric loss. The pronounced tunability observed in the dielectricity and piezoelectricity (Figure 2d,c) naturally leads one to question the extent to which dielectric loss and AC conductivity might be impacted by dislocation imprint. To address this, we assessed the dielectric loss and AC conductivity in both reference and deformed samples. In **Figure** **3**a, one can see that the AC conductivity of both D1300 and N1150 samples increased by more than two orders of magnitude after deformation as compared to the reference sample. As anticipated, the D1150 sample demonstrated the highest increase in AC conductivity by about a factor of 600, in fact. For the deformed samples with different dislocation densities, the predominant contribution arises from the increase in dielectric loss (Figure 3b). Conversely, a secondary, albeit still significant, contribution stems from the increase in dielectric permittivity, as illustrated in Figure 3b. Therefore, 1D‐2D defect interactions are primarily linked to the significantly enhanced dielectric loss of the deformed sample. However, the increase in dielectric response is more related to the mobility of the domain walls, that is to say, dislocations with a high density could severely pin the motion of domain walls and limit the mobility of these walls, leading to a reduced dielectric response. These dislocation density‐tuned dielectric properties lead to the expected importance of the control of the AC conductivity in bulk ferroelectrics. Note that the significant subcoercive dielectric loss contributes to a minor thermal expansion, representing ≈1% of the measured piezoresponse of the deformed sample.^[^ ^61^ ^]^

### 2.3 Origin of Dislocation Density‐Tuned Functionality

**Figure 4:**
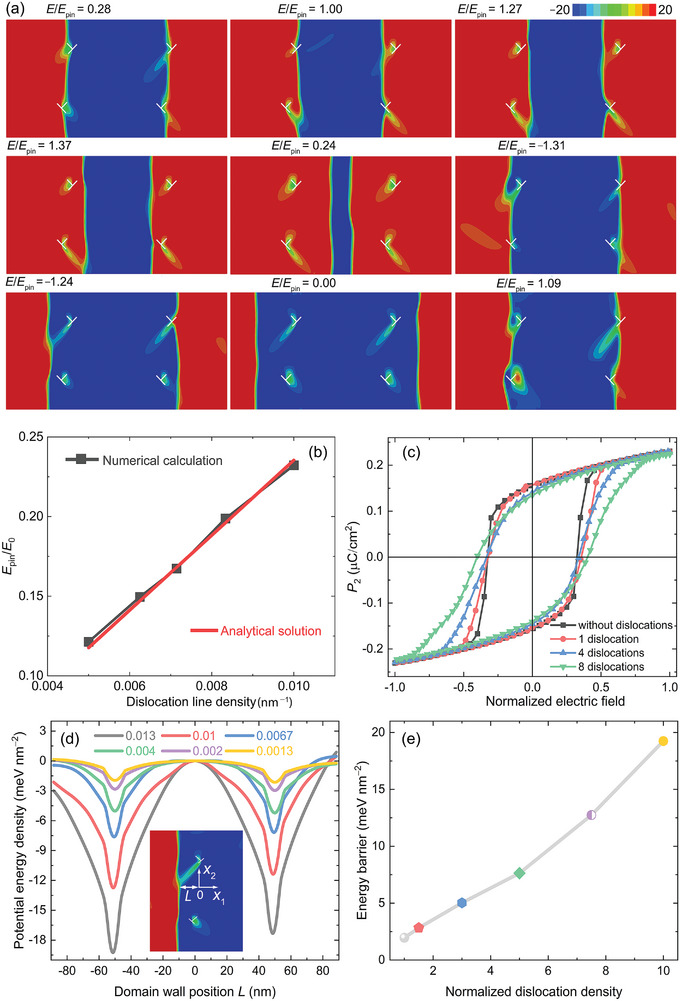
a) The dislocation arrays can pin the motion of domain walls and ensure reversible displacement of the domain walls. The unit for the polarization is µC cm^−2^. b) Analytical and numerical calculations of the pinning field, *E*_pin_, as a function of dislocation density. The calculated *E*_pin_ is normalized to *E*_0_ = 9.56 kV mm^−1^. c) Simulated polarization hysteresis loops of BaTiO_3_ single crystals without and with different dislocations. The applied field is normalized to *E*_0_. d) The potential energy density as a function of the domain wall position (*L*) at different dislocation densities. The inset shows the left half side of the numerical model for computing driving force and potential energy on the domain wall induced by two dislocations with perpendicular Burgers vector. e) The potential energy barrier is proportional to the dislocation density. The dislocation density is normalized to 0.0013 nm^−1^.

To acquire a more profound comprehension of the relationship between dislocation density and functional response, we provided a mechanistic rationale for the intricate and surprising interactions between dislocations and domain walls and constructed a physical depiction of domain wall dynamics utilizing a large set of phase‐field simulations. Our phase‐field model in detail can be found in the Experimental section. When the imprinted 1D dislocations align with the 2D domain walls (Figure S19a, Supporting Information), the local stress fields (in the order of GPa) in the vicinity of these dislocations cause the wall to curve outward, specifically toward the tensile side of the dislocation (Figure S19b, Supporting Information). Note that the intricate dislocation networks are embedded into 90° domain walls, adhering to the parallel configuration. The strain hardening during high‐temperature plastic deformation indicates multi‐slip structures of dislocations with perpendicular Burgers vector.^[^ ^17^ ^,62]^ To highlight the key feature of the high‐temperature uniaxial compression activated {100}<100> slip systems, we simulated the interactions between four dislocations with perpendicular Burgers vector and two domain walls, as depicted by our model adopted in **Figure** **4**a. This simple dislocation network serves as a didactic demonstration to elucidate the behavior of both pinning and depinning (see Video S1, Supporting Information). When the applied field is <*E*_pin_ (0.28*E*_pin_ in Figure 4a), the domain walls are entirely pinned by dislocations, and begin to depin only when the field escalates to reach *E*_pin_. The domain walls get fully depinned once the applied field is higher than 1.37*E*_pin_. A similar phenomenon of pinning and depinning is obtained when a negative field is applied. To obtain a clearer picture of the effect of dislocations on the *E*_pin_, we computed the dependence of *E*_pin_ on dislocation density. Our numerical calculations and analytical solution revealed that *E*_pin_ increases linearly with increasing dislocation density (Figure 4b). Furthermore, the introduction of dislocations significantly reduces the polarizations (both remanent polarization and maximum polarization) but enhances the coercive electric field on the macroscopic level (see our simulated polarization hysteresis loops in Figure 4c), which is in good agreement with our experimental results (Figure 2a). In order to simulate polarization hysteresis loops, we utilized the phase field models' sets with varying dislocation density (Figure S20, Supporting Information).

The behavior of domain walls is directly related to the electromechanical properties of ferroelectrics, which is attributed to their changes in potential energy when bypassing a dislocation. For the model with four dislocations with perpendicular Burgers vectors, as depicted in Figure 4a, the calculated potential energy profiles of 90° domain walls indicate that the four dislocations formulate two deep potential energy wells (Figure 4d). Based on our TEM observations, we covered the dislocation density over one order of magnitude. Two dislocations with perpendicular Burgers vectors provide an antisymmetric driving force on the domain wall between them (Figure S21, Supporting Information). The slope of the driving force between the two dislocations is negative, which indicates the existence of a local minimum potential energy state of the domain wall, as revealed in Figure 4d. The driving force and multiple potential energy wells with different dislocation densities feature in the same manner and enable reversible and irreversible displacements of the domain walls, which contribute to the electric field dependence on (extrinsic) electromechanical properties.^[^ ^63^ ^]^ Essentially, when moving from one potential well into another one, the pinning and depinning of the domain wall are the origin of the dielectric loss. Interestingly, akin to the dependence of *E*_pin_ on dislocation density, the potential energy barrier is also proportionate to the dislocation density, as plotted in Figure 4e. With a higher dislocation density and a larger energy barrier, it is more difficult for the domain wall to irreversibly displace, leading to reduced mobility of the domain wall. As a result, the extrinsic contributions to the electric field‐dependent AC conductivity, dielectric, and electromechanical properties are discouraged. Our calculated results emphasize the influence of dislocation density on domain wall pinning and the nonlinear piezoelectric and dielectric response, thereby contributing to intrinsic variations in piezoelectric and dielectric properties, as illustrated in Figure S22, Supporting Information. Given our phase‐field model simulates a simple dislocation network within a limited sample size (see methods), it is incapable of replicating the experimentally observed dielectric and piezoelectric response without a deeper understanding of the complex interplay between dislocations and domain walls.

## 3 Conclusion

Due to the brittleness of most ferroelectric oxides, engineering dislocations into bulk ferroelectrics remains one of the most pressing bottlenecks. We revealed that plastic‐deformation‐induced dislocation structures with controlled density can be effectively utilized to achieve optimized electromechanical response and control the mobility of ferroelectric domain walls in single‐crystal BaTiO_3_. While the dislocation density was altered by approximately an order of magnitude, we were able to significantly tune the piezoelectric and dielectric properties, and even enhance the AC conductivity by two orders of magnitude. This dislocation‐density‐based plastic strain engineering, akin to the elastic strain engineering and an alternative to the existing chemical doping approach, may provide us with superb control of the piezoelectricity, dielectricity, and conductivity in ferroelectric systems with non‐180° and 180° domain walls. Further advancement will depend on expanding the deformation techniques developed for prototypical BaTiO_3_ to encompass other ferroelectric ceramics. Preliminary unpublished work demonstrated that dislocation structures can be imprinted into large grain‐sized polycrystalline SrTiO_3_. Also, polycrystalline SrTiO_3_ could be furnished already with dislocations by high‐temperature deformation.^[^ ^64^ ^]^ For example, managing the dislocation density at or close to the interface between thin‐film ferroelectrics and the substrate could herald a novel methodology for tuning functional oxides.^[^ ^49^ ^,65]^ Considering the ubiquitous nature of dislocations in crystalline solids, the significant influence of dislocation density on functional properties could have extensive implications across a diverse range of material systems that couple to dislocation‐type strain, such as antiferromagnetic NiO,^[^ ^66^ ^]^ multiferroic BiFeO_3_ and non‐ferroelectric SrTiO_3_.^[^ ^18^ ^,67]^ Thus, dislocation engineering could potentially pave the way for a wide array of novel functional materials.

## 4 Experimental Section

### Sample Preparation

Dislocations with {100}<100> slip planes were introduced by high‐temperature plastic deformation in [110]‐oriented BaTiO_3_ single crystals (4 × 4 × 8 mm^3^, coordinate system: *X*: $\bar{[1}10]$; *Y*: [001]; *Z*:[110], Electro‐Optics Technology GmbH). The fundamental nature of the dependencies of the dislocation density on experimental loading parameters such as stress, strain, and temperature has been discussed in the textbook.^[^ ^68^ ^]^ Based on the previous work,^[^ ^53^ ^]^ it was found that the yield strength of [110]‐oriented BaTiO_3_ single crystals mainly depends on temperature. Uniaxial compression experiments along the [110] direction at 1150 °C were performed using a load frame (Z010, Zwick GmbH) equipped with an HTM Reetz furnace (HTM Reetz GmbH). A linear variable differential transducer (LVDT) allowed to quantify precise displacements. Similar loading experiments at 1300 °C were conducted using a load frame (Instron, USA) furnished with an HTM Reetz furnace and an LVDT. A pre‐load of 20 N was applied during heating and cooling with a ramp of 1 °C min^−1^. The sample was held at the targeted temperature (1150 °C or 1300 °C) for 30 min to reach thermal equilibration. BaTiO_3_ single crystals were deformed with a loading rate of 0.2 N s^−1^ up to an engineering strain of ≈2% in compression and then were unloaded with a rate of 0.5 N s^−1^. Several samples featured barreling after deformation (Figure 1a), so all deformation experiments were stopped in the relatively low percentage range of ≈2% in order to avoid microcracks. Experimental details for high‐temperature deformation can be found elsewhere in the literature.^[^ ^41^, ^47^, ^48^, ^53^ ^]^

In order to further increase the dislocation density, the single crystals were notched using a diamond wire saw (Well 4240, Well Diamantdrahtsägen GmbH).^[^ ^18^ ^]^ The notch was located in the middle of one of the (100) planes with a depth of 15%–25% of the thickness of the investigated sample. The notched crystals were subjected to uniaxial compression along the [110] direction at a temperature of 1150 °C using a Z010 load frame, employing the same loading conditions as the samples without a notch. This advanced mechanical deformation approach resulted in the formation of a highly dense and localized dislocation field along the {100}<100> slip bands (Figure 1a).

Small slices along the [110] and [001] directions were extracted for structural and electrical characterization (Figure S2, Supporting Information). To minimize the impact of heat treatment, the reference samples underwent identical heat treatment procedures as the D1150 and N1150 samples. The surface of the slices was g round using sandpaper to achieve plane‐parallel surfaces. The crystallographic orientations of the [110]‐ and [001]‐oriented samples were confirmed using a 1001 Model Laue back reflection with an accuracy of ±0.5°. These sliced samples were polished with a series of mechanical polishing steps with a polishing machine (Phoenix 4000, Jean Wirtz GmbH), followed by 6 µm particle size (30 min), 3 µm (30 min), 1 µm (30 min) and 0.25 µm (30 min). Samples for scanning electron microscopy were additionally treated using vibration polishing (Jean Wirtz, KAWA GmbH) for ≈20 h. For electron channeling contrast imaging, the samples were sputtered with a thin layer of carbon. The dimensions for the electrical measurements were ≈4 mm × 4 mm × 1 mm. Gold electrodes were sputtered on the two large {110} faces for electrical characterization with an electric field applied in the [110] direction. All investigated samples were annealed at 200 °C for 2 h.

### Dislocation and Domain Structure Characterization

Optical images were recorded using a LEXT laser scanning microscope (OLS4100, Olympus). Both polarized light mode and differential interference contrast (DIC) mode were used to visualize domain patterns. PFM measurements were conducted with a Cypher atomic force microscope (Asylum Research, USA) using conductive Cr/Pt‐coated silicon cantilever tips, which have a spring constant of ≈3 N m^−1^ and a resonance frequency of ≈75 kHz. During the overview PFM scans, a modest AC driving voltage of 1 V (peak amplitude) was applied. X‐ray diffraction (XRD) experiments were conducted on [001]‐oriented samples using a Bruker D8 diffractometer (Bruker Corporation, Germany) in Bragg‐Brentano geometry with Cu‐Kα_1,2_ radiation. To obtain the electron channeling contrast images of the samples, a low tilt configuration consisting of a 4‐quadrant solid state back‐scattered electron detector (DEBEN, UK) mounted under the polepiece of a field emission scanning electron microscope (FE‐SEM, MIRA3‐XMH‐TESCAN) was used. To avoid charging the surface, an extremely thin layer of carbon was coated on the specimens. A high voltage (20–25 kV) was utilized to eliminate any possible surface effects. The dislocations and the dislocation line can be visualized as white spots and the following line shadow behind it, respectively. To have the highest contrast the samples were rotated and tilted until the incoming electrons could channel through the crystal and the backscattered signal related to dislocations was maximized.

For high‐resolution imaging, the deformed crystals were cut into small pieces with 300‐µm thickness and polished using a MultiPrep polishing system (Allied High Tech Products Inc., USA) down to a thickness of 20 µm. Followed by half an hour of annealing at 200 °C, the annealed slices were mounted on supporting molybdenum grids of 100 mesh (Plano, Germany) and thinned by an Ar‐ion beam using a dual ion milling system (Gatan, USA) into electron transparency. Bright‐field images were taken using a TEM (JEM‐2100F, JEOL) operated at 200 kV.

^137^Ba solid‐state NMR spectra were recorded using a spectrometer (Avance III HD, Bruker) equipped with a 14.1 T Oxford wide bore magnet. A single‐axis goniometer NMR probe (NMR Service, Erfurt) with a nominal resolution of 0.1° was tuned to 66.71 MHz. NMR spectra at given angles were obtained by exposing a (110) face. An angle of 0° represents the normal vector of the sample holder as parallel to the magnetic field B_0_. A 90°−180° Hahn‐echo sequence with τ = 30 µs, a recycle delay time of 1 s, and an acquisition time of 0.05 s was employed. The duration of the 90° pulses was set to 3.5 µs and the number of scans to 10 240. The scale of the chemical shift was referenced to a 1 m solution of BaCl_2_ (0 ppm).

### Electrical Measurements

Temperature‐dependent dielectric permittivity was quantified with an applied AC field of 1 V using an impedance analyzer (HP 4192 A, Hewlett Packard) equipped with a furnace (Nabertherm GmbH) with a heating ramp of 1 °C min^−1^. Polarization hysteresis loops at room temperature were recorded at 1 Hz using a ferroelectric test system (TF 2000E, aixACCT Systems Inc.). To simultaneously quantify the sub‐coercive field dependence of converse piezoelectric coefficient (*d*_33_*) and permittivity (*ε*_33_), the driving‐voltage amplifiers and dielectric analyzer were combined with a laser vibrometer (OFV‐505 Sensor Head and VDD‐E‐600 Front‐End, Polytec GmbH). Self‐developed Macro codes were used to pick up the developed displacements of the investigated sample.

### Phase‐Field Simulations

A phase‐field model combined with a non‐singular continuum theory of dislocations was adopted to simulate the interaction between domain walls and dislocations in the plastically deformed BaTiO_3_ single crystals.^[^ ^26^, ^69^, ^70^ ^]^ The spatial distribution of spontaneous polarization vector *P*(*P*_1_, *P*_2_, *P*_3_) was used to describe the evolution of the domain structure. The coupled electro‐mechanical problems were governed by the mechanical stress equilibrium equation, Gauss's law, and the time‐dependent Ginzburg–Landau equation:

(2) $$\sigma_{ij,j}=\;0$$

(3) $$D_{i,i}-q\;=\;0$$

(4) $${\dot{P}}_{i}=-M\frac{\delta F}{\delta P_{i}}$$

where *q* is the volume charge density, ${\dot{P}}_{i}$ is its time derivate, *M* refers to the mobility parameter and F is total free energy of the system, respectively. The stress σ_*ij*_ and electric displacement *D_i_* were defined through the constitutive relations:

(5) $$\sigma_{ij}=\frac{\partial f}{\partial \varepsilon_{ij}}\;=c_{ijkl}\;\left(\varepsilon_{kl}-\varepsilon_{kl}^{P}-\varepsilon_{kl}^{D}\right)$$

(6) $$D_{i}=\;-\;\frac{\partial f}{\partial E_{i}}=K_{ij}\;E_{j}+P_{i}$$

where *c_ijkl_* were the elastic stiffness tensors,  ε_*ij*_ =  (*u*_*i*,*j*_ + *u*_*j*,*i*_)/2 is the total strain defined as the symmetric part of the displacement gradients *u*_*i*,*j*_. $\varepsilon_{ij}^{P}=Q_{ijkl}\;P_{k}P_{l}$ is the eigenstrain induced by polarization with *Q_ijkl_* as the electrostrictive coefficients. The dielectric constants were defined as *K_ij_* = ω_0_ κδ_*ij*_ with the dielectric permittivity of vacuum ω_0_, the relative dielectric permittivity of the background of the bulk κ, and the Kronecker delta  δ_*ij*_. The electric field is defined as *E_i_* =   − φ_, *i*_, where φ is the electric potential. The eigenstrain of dislocations, $\;\varepsilon_{ij}^{D}\left(x\right)$ is formulated based on a non‐singular continuum theory of dislocations:^[^ ^26^, ^70^ ^]^

(7) $$\varepsilon_{ij}^{D}\;\left(x\right)=\frac{1}{2}\;\left(b_{i}n_{j}+b_{j}n_{i}\right)\int_{S}\tilde{w}\left(∥x-x^{D}∥,h\right)dS\left(x^{D}\right)$$

where *b_i_* is the Burgers vector, *n_i_* is the normal vector, *h* defines the dislocation core size, and $\tilde{\;w}\left(x,h\right)$ is the spreading function of the Burgers vector around the slip plane.^[^ ^70^ ^]^ The total free energy of the system is given by

(8) $$\begin{array}{ccc} F & = & F_{bulk}\;\left(P\right)+F_{elas}\left(\varepsilon ,P\right)+F_{elec}\left(P,E\right)+F_{grad}\;\left(P\right)\\ & = & \int_{V}f_{bulk}+f_{elas}+f_{elec}+f_{grad}\;dV \end{array}$$

The bulk Landau free‐energy density is expressed as an eighth‐order polynomial expansion, namely,

(9) $$\begin{array}{ccc} f_{bulk} & = & \alpha_{ij}\;P_{i}P_{j}+\alpha_{ijkl}P_{i}P_{j}P_{k}P_{l}+\alpha_{ijklmn}P_{i}P_{j}P_{k}P_{l}P_{m}P_{n}\\ & & +\alpha_{ijklmnpq}P_{i}P_{j}P_{k}P_{l}P_{m}P_{n}P_{p}P_{q} \end{array}$$

where α_*ij*_, α_*ijkl*_, α_*ijklmn*_ and α_*ijklmnpq*_ were the Landau‐Devonshire coefficients. The gradient energy density can be written as $f_{grad}=\frac{1}{2}\;g_{ijkl}P_{i,j}P_{k,l}$, where *g_ijkl_* were the gradient coefficients. The electrostatic energy density can be described as $f_{elec}=\;-\frac{1}{2}K_{ij}E_{i}E_{j}-P_{i}E_{i}$. The elastic energy density can be expressed as $f_{elas}=\frac{1}{2}\;c_{ijkl}\left(\varepsilon_{ij}-\varepsilon_{ij}^{P}-\varepsilon_{ij}^{D}\right)\left(\varepsilon_{kl}-\varepsilon_{kl}^{P}-\varepsilon_{kl}^{D}\right)$.

The computation of driving forces on the domain wall was based on generalized configurational mechanics of dislocations in ferroelectrics,^[^ ^69^ ^]^ where the driving force is defined as:

(10) $$F_{k}=\int_{V}\Sigma_{kj,j}dV$$

with the Eshelby stress tensor

(11) $$\Sigma_{kj}=\;f\delta_{kj}-\sigma_{ij}\beta_{ik}+D_{j}E_{k}-g_{ijmn}P_{m,n}P_{i,k}$$

where β_*ik*_ = *u*_*i*,*k*_  − *b_i_n_k_W*(**x**,*h*). The fully coupled electromechanical problems were solved in Equations ((2), (3), (4)) simultaneously using the finite element method. In the finite element simulations, the driving force is calculated in the postprocessing (see^[^ ^69^ ^]^ for technical details).

(12) $$F_{k}=\;-\sum_{I\;=\;1}^{n_{nod}}G_{k}^{I}$$

(13) $$\;G_{k}=\overset{n_{el}}{\underset{e=1}{\cup }}\int_{V_{e}}\Sigma_{kj}N_{,j}^{I}dV$$

Here *n_nod_* is the number of nodes in the integration domain, *G_k_* is the nodal configurational force, *V_e_* is the volume of the element, *n_el_* is the number of elements around a node, and $N_{,j}^{I}$ is the gradient of the shape function in the finite element method. With the computed driving force on the domain wall, the potential energy of the domain wall is then calculated as:

(14) $$U\;=\;-\int_{L^{1}}^{L^{2}}F_{1}^{P}dx_{1}$$

where$\;F_{1}^{P}$ is the driving force on the domain wall. *x*_1_ in the range from *L* ^1^ to *L* ^2^ is the position of the domain wall in the horizontal direction.

The numerical simulations analyze the interaction between the 90° domain wall and the dislocation. The material parameters can be found^[^ ^71^ ^]^ and Table S1, Supporting Information. Four simulations were performed. The first simulation provides the pinning and depinning of the domain wall under an external electric field, as featured in Figure 4a. The sample size was 200 × 100 nm^2^ with mesh size of 1 nm. The dislocations were located at coordinates (−65, −25), (−55, 25), (55, −25), and (65, 25). The periodic boundary condition was applied for all boundaries. The second simulation was the benchmark for the dependence of *E*_pin_ on dislocation density, as depicted in Figure 4d. The sample size was 100 × *H* nm^2^, where *H* was the sample height and 1/*H* was the dislocation line density. The periodic boundary condition was applied for the top and bottom boundaries. The left and right boundaries were traction‐free, and the applied surface charge density was consistent with *P*_1_. *E*_pin_ was determined by the critical electric field for the domain wall to bypass one dislocation located at the center of the sample. The third simulation computes the *P*− *E* hysteresis loop, as revealed in Figure 4c. The sample size was 60 × 60 nm^2^. The fourth simulation computes the dependence of the potential energy line density (*U*/*H*) of the domain wall on the dislocation density. The sample size was 100 × *H* nm^2^. Two dislocations with perpendicular Burgers vectors were located at coordinates (−5, *H*/4) and (−5, *H*/4). For simplicity, the influence of dislocations was assumed on the domain wall was negligible when the domain wall was far away from dislocations, and thus two potential energy wells were constructed by superposition of the calculated driving forces of two dislocations with shifted coordinates ($F_{1}\;\left(x_{1}^{\prime }\right)=F_{1}\;\left(x_{1}+50\right)+F_{1}\left(x_{1}-50\right)$). The phase field model was numerically implemented using the finite element method within the simulator “Panda's Multi‐Physics” (bitbucket.org/pandasmultiphysics/pmp/wiki/Home) developed based on the open‐source software Multiphysics Object‐Oriented Simulation Environment (MOOSE) framework.^[^ ^72^ ^]^ Numerical simulations were carried out on the high‐performance cluster at the Department of Mechanics and Engineering of Sichuan University.

## Conflict of Interest

The authors declare no conflict of interest.

## Author Contributions

F.Z. and X.Z. contributed equally to this work. J.R. and F.Z. conceived the original idea. F.Z. carried out deformation experiments, J.R. and F.Z. discussed the deformation mechanisms. F.Z. prepared the samples and measured the electrical properties, and J.R., F.Z., and X.Z. contributed to the electrical data analysis. F.Z. conducted XRD experiments and collected the optical and PFM images. X.Z. and B.‐X.X. performed the phase‐field simulations. M.S. collected the ECCI images. F.D. performed the NMR measurements with the guidance and analysis of P.B.G. and G.B. P.B. created the Macro codes within the VibSoft‐VDD software. J.R. supervised the project. F.Z. and J.R. wrote the manuscript. All authors participated in discussions and reviewed the paper.

## Supporting information

Supporting Information

Supplemental Video 1

## Data Availability

The data that support the findings of this study are available from the corresponding author upon reasonable request.
