# Supplementary material for: Dislocation Density‐Mediated Functionality in Single‐Crystal BaTiO3
Source: Adv Sci (Weinh). 2024 Jun 17;11(31):2403550. doi: 10.1002/advs.202403550 (PMC11336959; doi:10.1002/advs.202403550)
Supplement: Supplementary file 1 — Supporting Information [file ADVS-11-2403550-s001.docx]

Supporting Information

Dislocation density-mediated functionality in single-crystal BaTiO_3_

Fangping Zhuo*, Xiandong Zhou, Felix Dietrich, Mehrzad Soleimany, Patrick Breckner, Pedro B. Groszewicz, Bai-Xiang Xu, Gerd Buntkowsky, and Jürgen Rödel

F. Zhuo, M. Soleimany, P. Breckner, B.-X. Xu, J. Rödel

Department of Materials and Earth Sciences, Technical University of Darmstadt, 64287 Darmstadt, Germany

E-mail: zhuo@ceramics.tu-darmstadt.de

X. Zhou

Failure Mechanics and Engineering Disaster Prevention Key Laboratory of Sichuan Province, College of Architecture and Environment

MOE Key Laboratory of Deep Earth Science and Engineering, College of Architecture and Environment, Sichuan University, Chengdu 610065, China

F. Dietrich, G. Buntkowsky

Institute of Physical Chemistry, Technical University of Darmstadt, 64287 Darmstadt, Germany

P. B. Groszewicz

Department of Radiation Science and Technology, Delft University of Technology, Delft 2629JB, Netherlands

F.Z. and X.Z. contributed equally to this work.


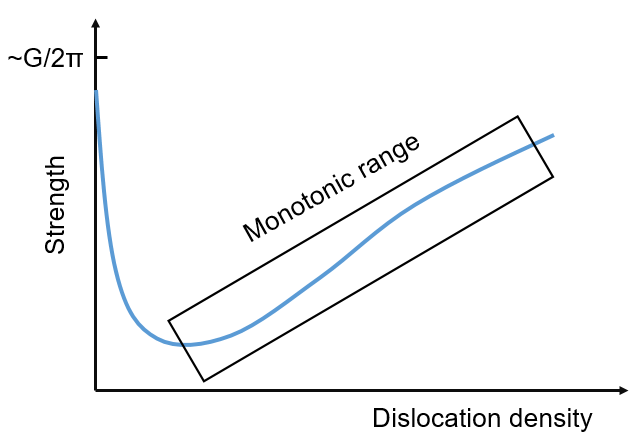


Figure S1. Schematic depiction of the relationship between the strength of metal crystals and density of dislocations.


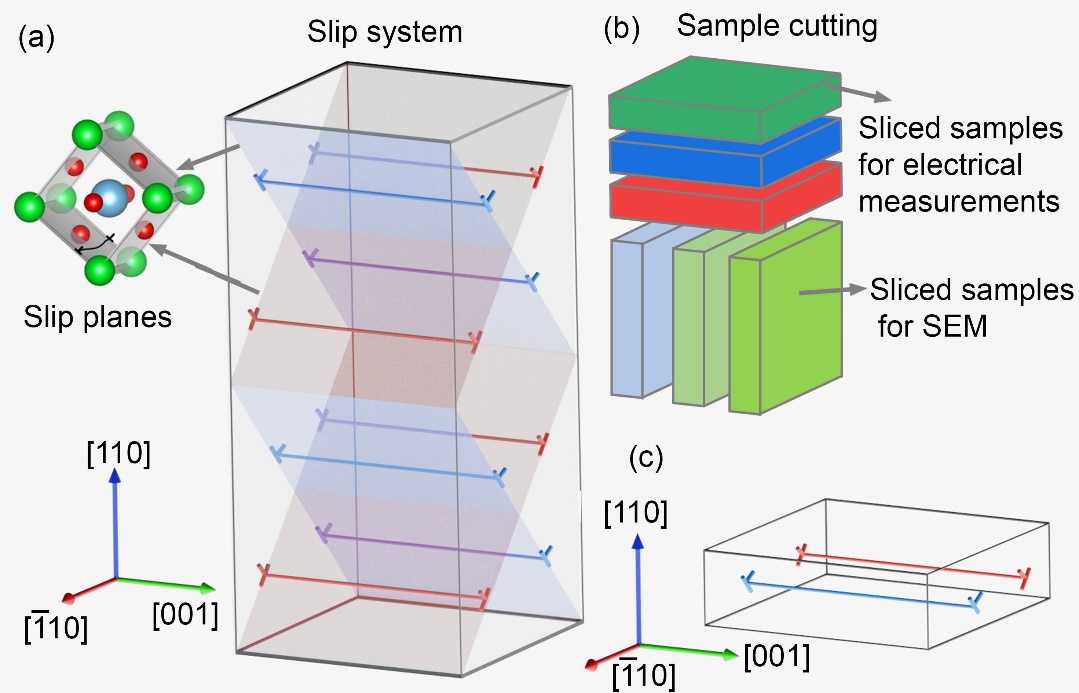


Figure S2. (a) Schematic illustration of the high-temperature {100}<100> slip systems. The sample was sliced into small pieces for electrical measurements and SEM/TEM experiments, as highlighted in (b). (c) Dislocation lines in the extracting slice for electrical measurements are perpendicular to the deformation direction [110].


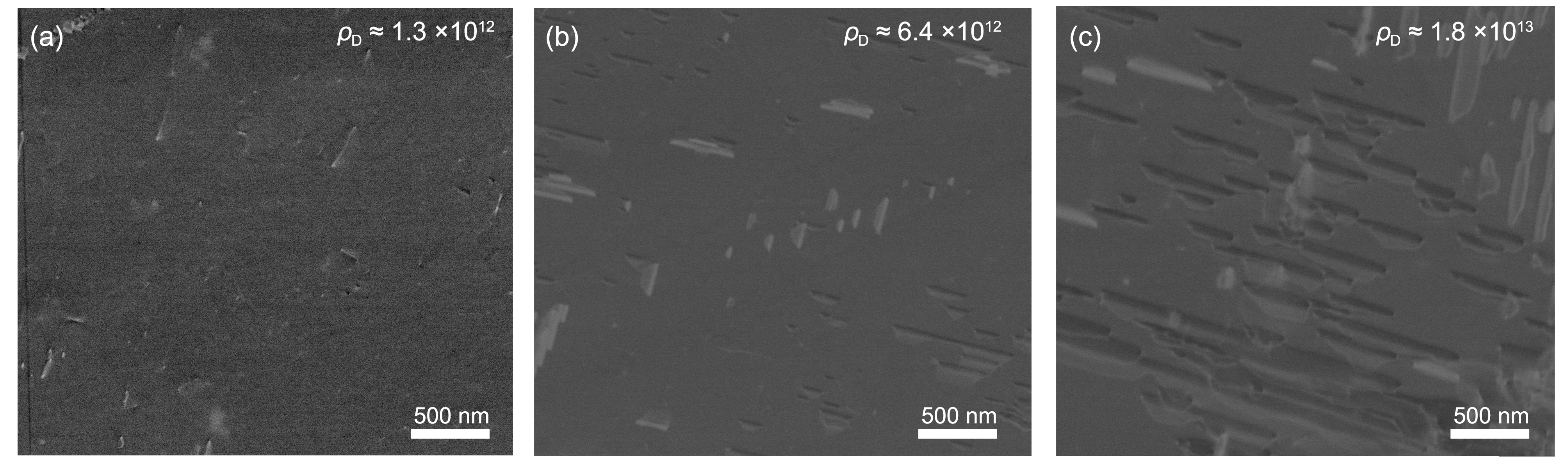


Figure S3. Representative electron channeling contrast imaging (ECCI) images of (a) D1150, (b) D1300 and (c) N1150. All ECCI images were viewed on the (001) plane. The locations of dislocations are marked as bright dots.


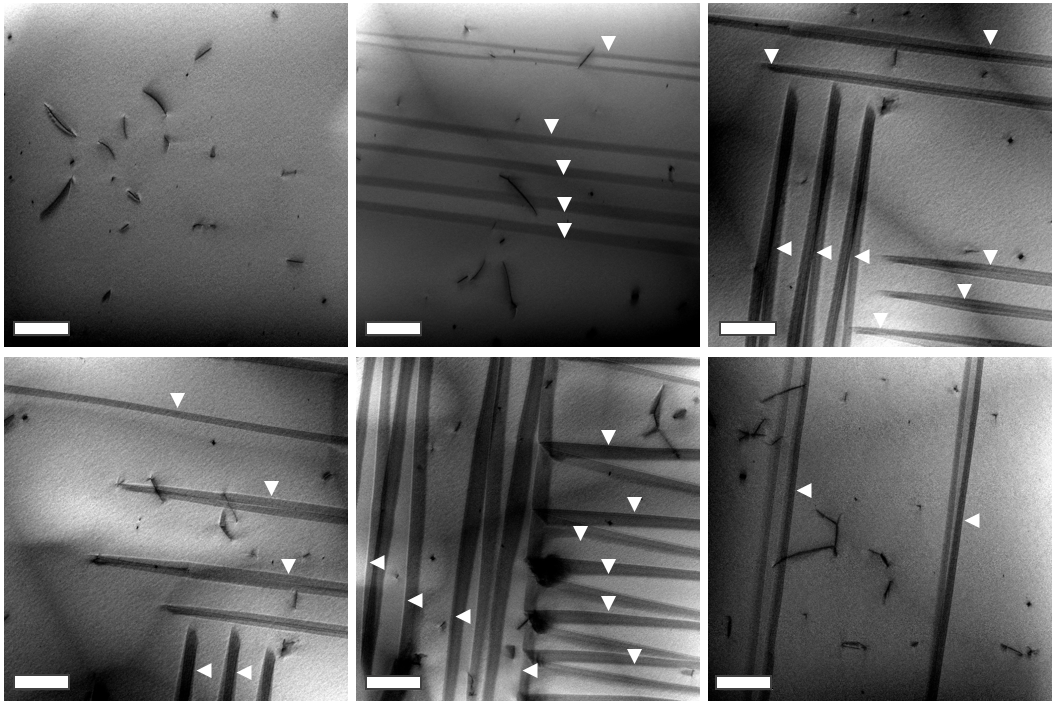


Figure S4. Bright-field TEM images taken from six different areas of the D1150 samples. The average dislocation density is determined to be 1.8 × 10^12^ m^–2^. All bright-field TEM images were viewed on the (001) plane. The positions of domain walls are marked by white arrowheads. Scale bars are 500 nm.


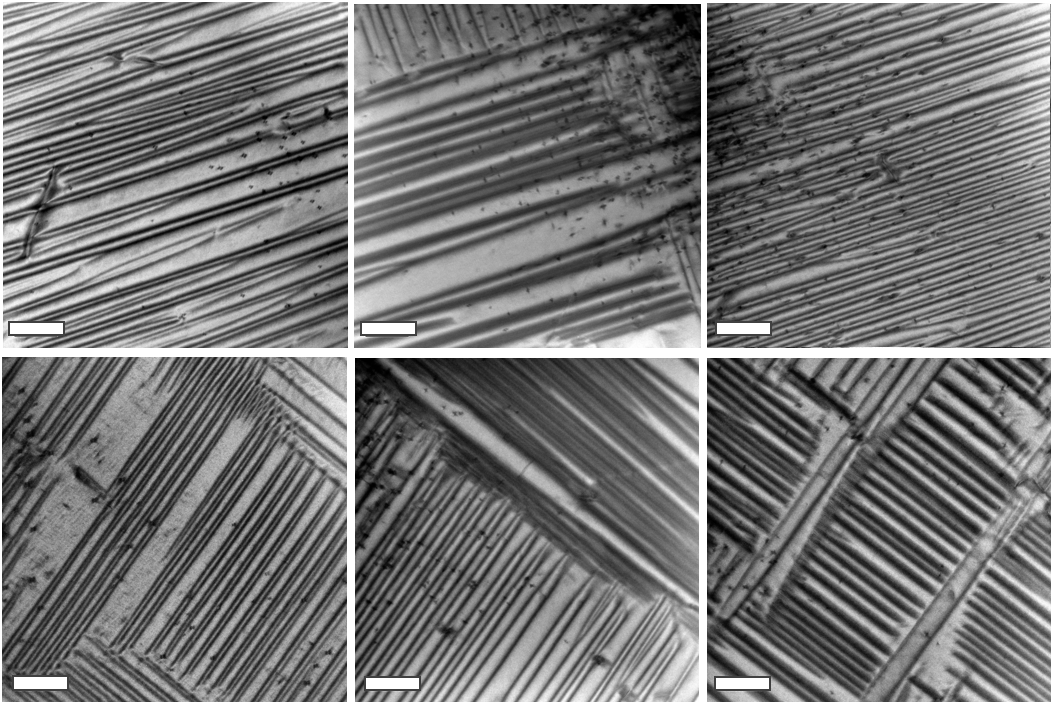


Figure S5. Bright-field TEM images taken from six different areas of the D1300 samples. The average dislocation density is determined to be 7.0 × 10^12^ m^–2^. All bright-field TEM images were viewed on the (001) plane. Scale bars are 500 nm.


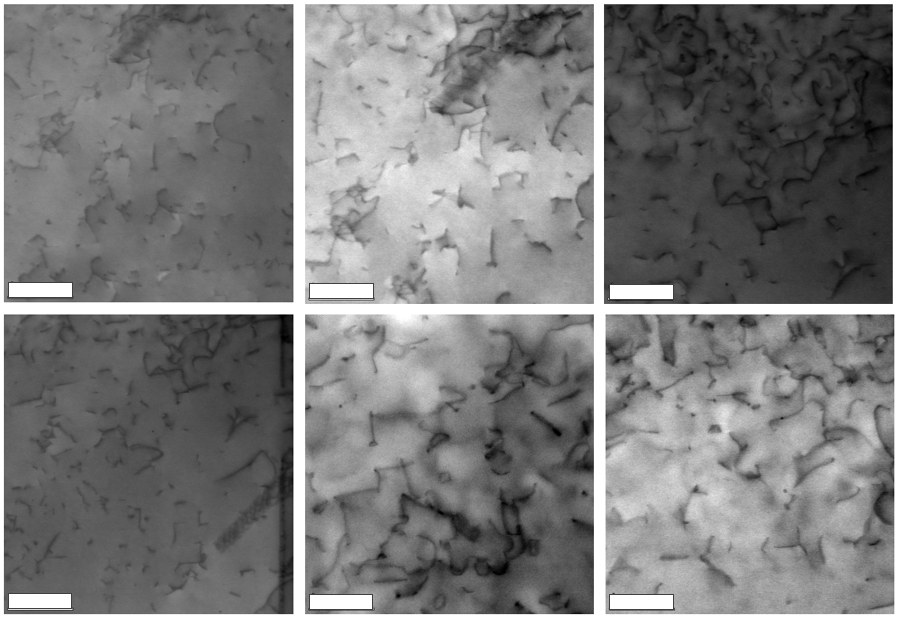


Figure S6. Bright-field TEM images taken from six different areas of the deformed sample with a notch. The average dislocation density is determined to be 1.5 × 10^13^ m^–2^. All bright-field TEM images were viewed on the (001) plane. Scale bars are 1000 nm. The absence of domain walls in these images may be attributed to the large domain size, as evidenced by our optical images in Figure S10.


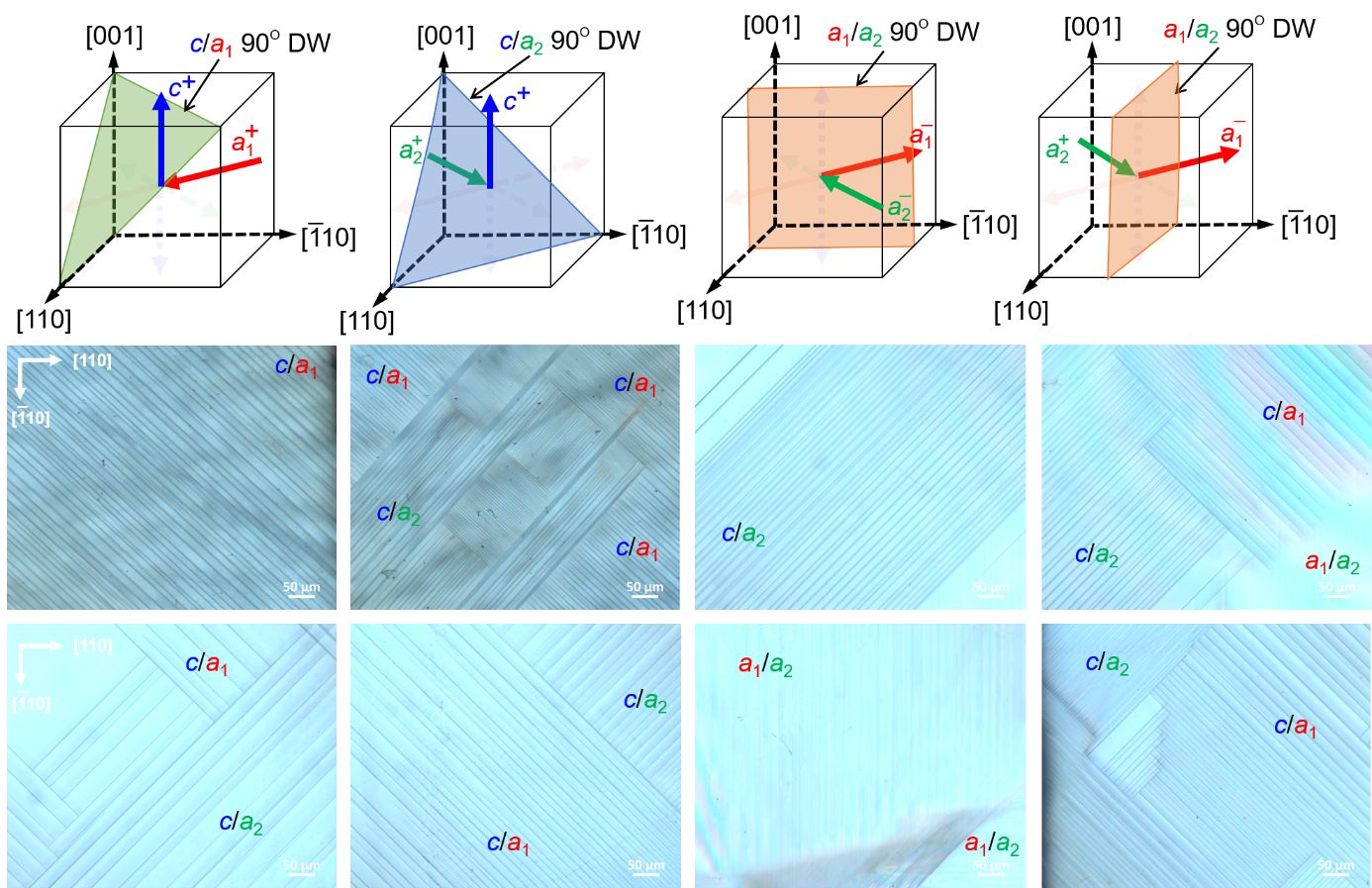


Figure S7. Optical images taken from eight different areas of the reference sample. The average domain size is determined to be ~24 μm.


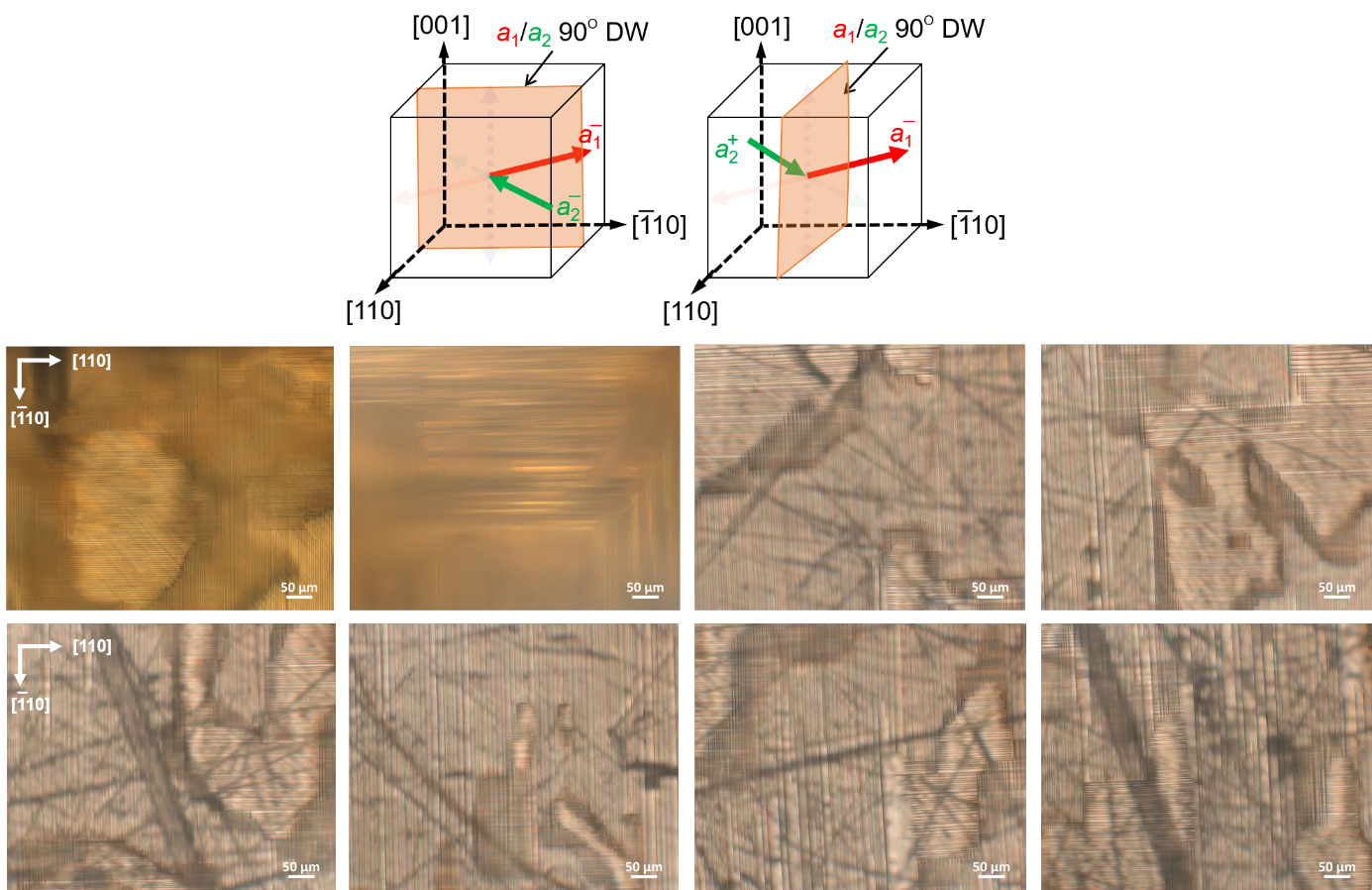


Figure S8. Optical images taken from eight different areas of the D1150 sample. The average domain size is determined to be 8 μm.


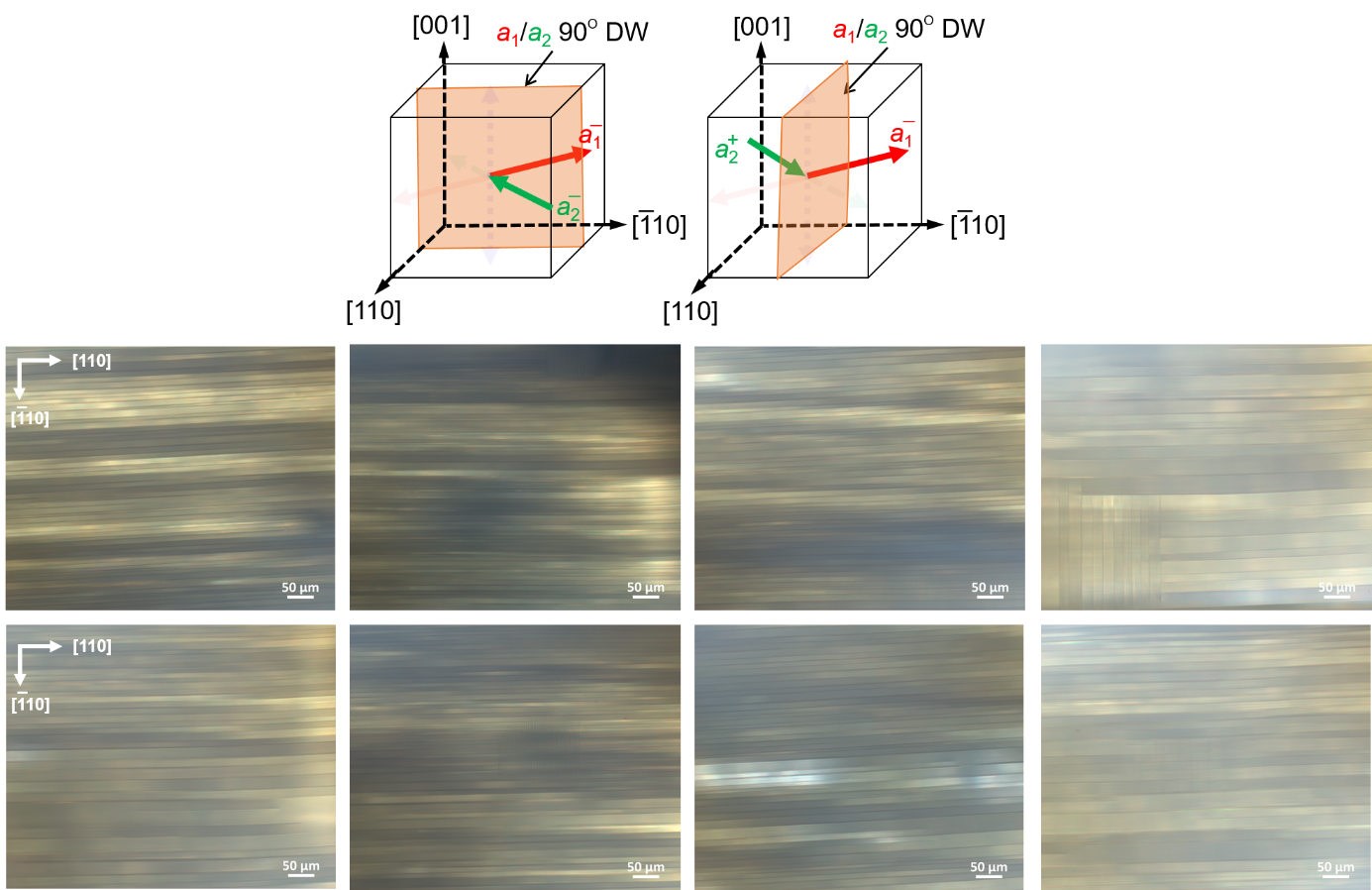


Figure S9. Optical images taken from eight different areas of the D1300 sample. The average domain size is determined to be 20 μm.


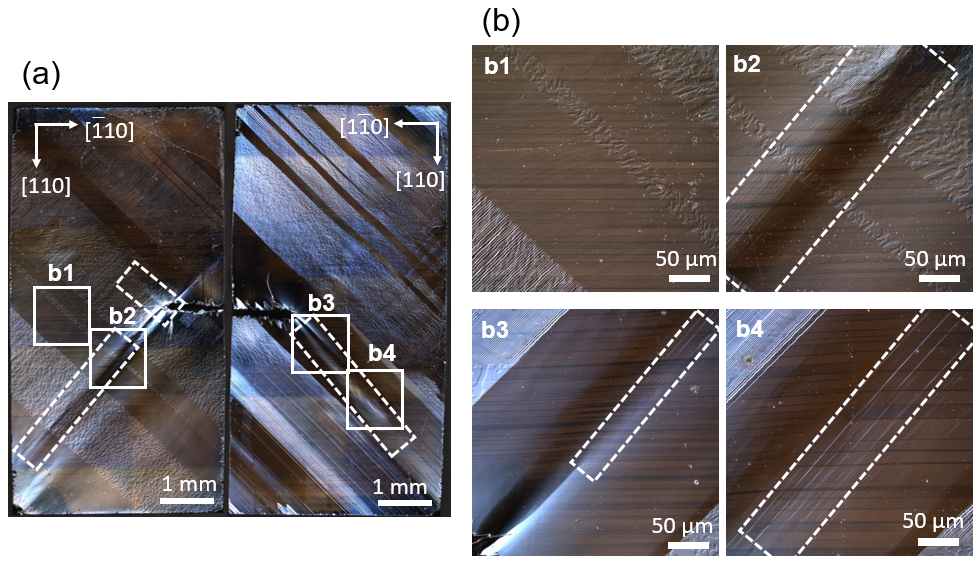


Figure S10. (a) After uniaxial deformation of a BaTiO_3_ single crystal in [110] direction with a notch inserted, slip traces (marked by dashed boxes) with a 45° angle with respect to the [110] direction can be seen. (b) Enlarged areas from both side surfaces marked by solid boxes in (a). The dislocation density in the slip traces is expected to be high, leading to the bending of the 90° domain walls. It is important to note that the coffee-bean-like features observed on the surfaces were a result of chemical polishing. The average domain size is determined to be 65 μm.


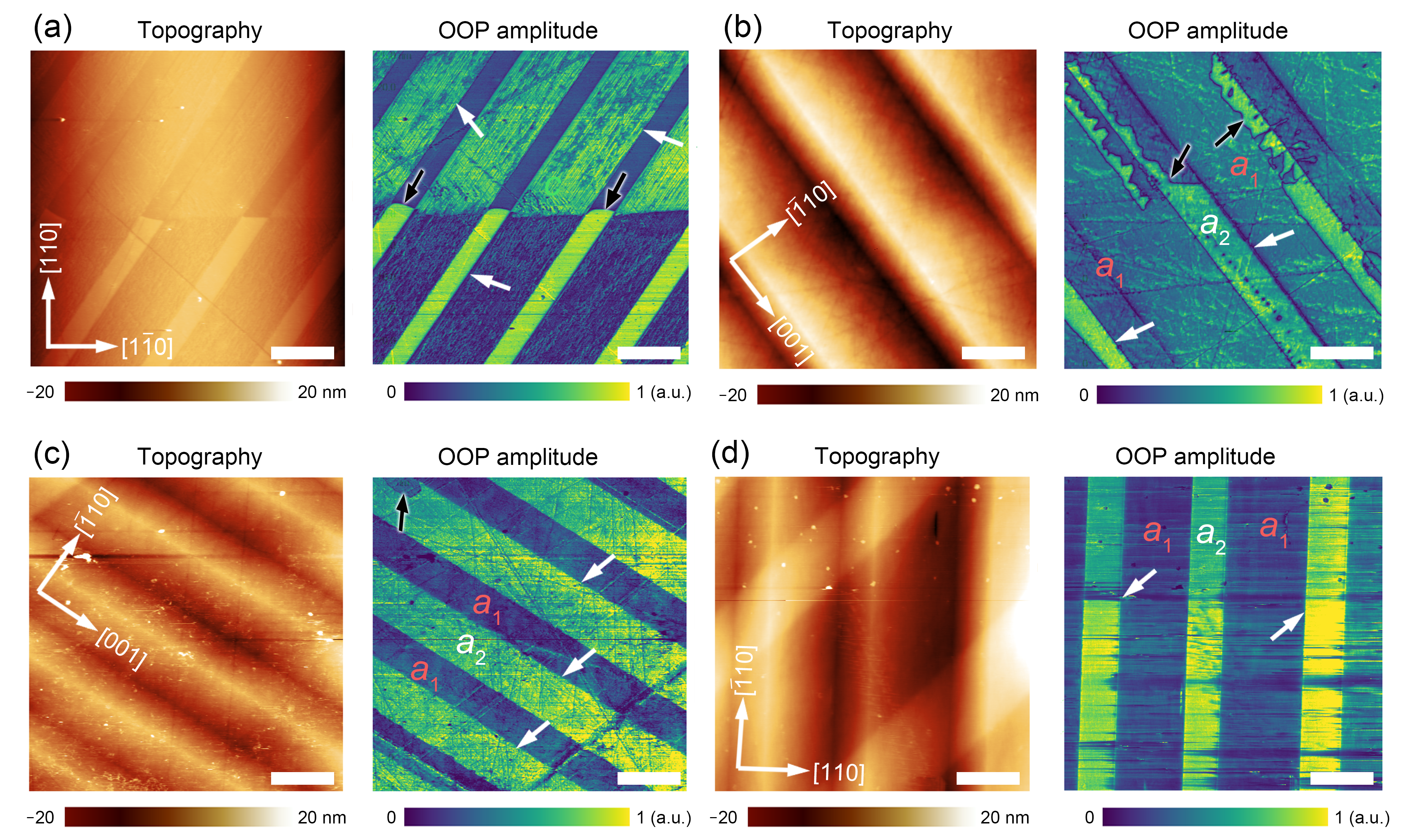


Figure S11. PFM images of (a) reference undeformed sample, (b) D1150, (c) D1300 and (d) N1150. The positions of 180° and 90° domain walls are marked by black and white arrows, respectively. The scale bar is 5 μm.


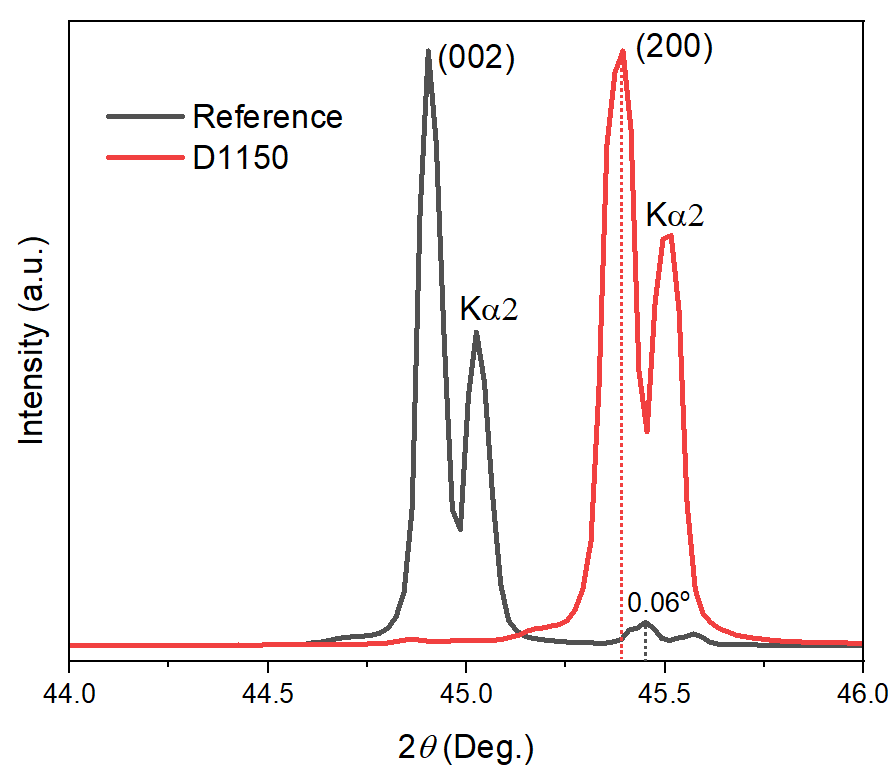


Figure S12. XRD patterns of the reference and deformed (D1150) samples. According to Bragg’s Law, a shift of 0.06° for the (200) peak corresponds to a change of about 0.26‰ in the lattice parameters.


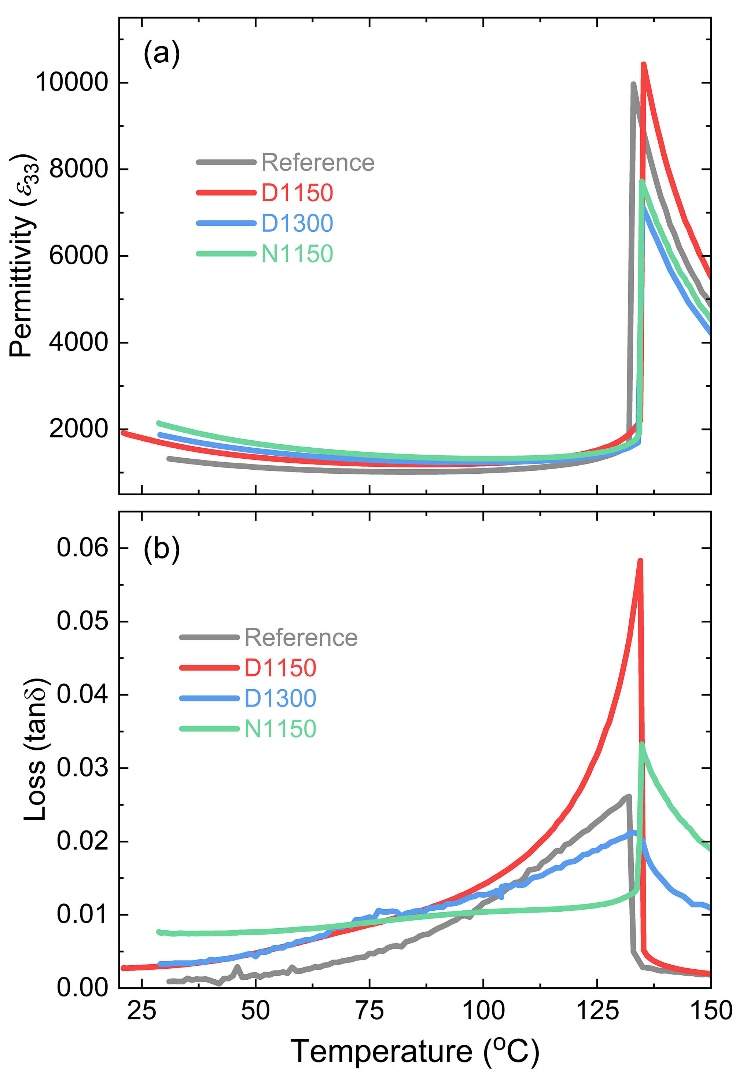


Figure S13. Temperature dependence of small-signal dielectric permittivity (a) and loss (b) at 1 kHz for reference and deformed samples.


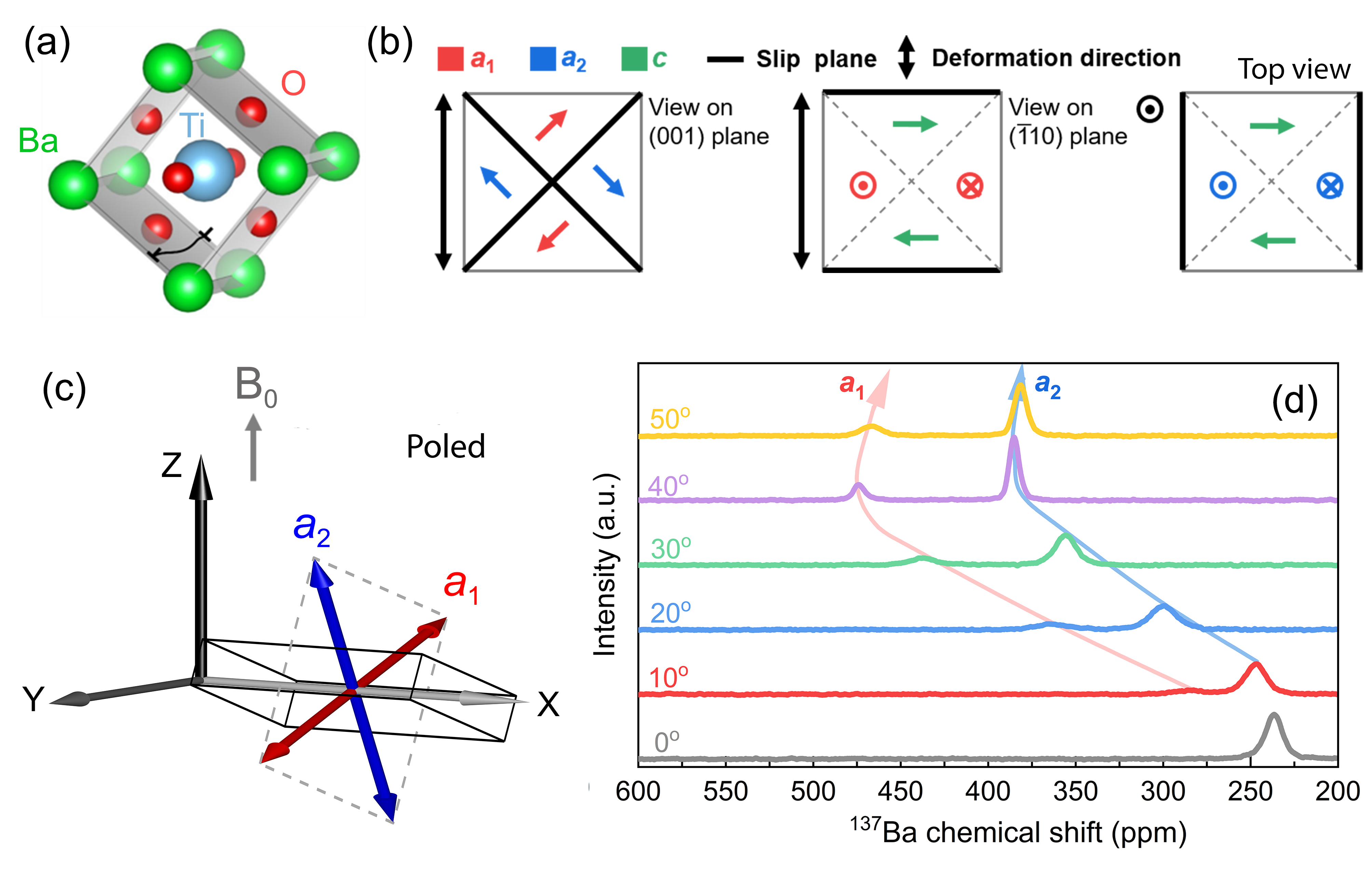


Figure S14. (a) Schematic highlighting the four slip planes of the high temperature <100>{100} slip system of BaTiO_3_ with Schmid factor *m* = 0.5 (marked with gray color) which are activated during uniaxial compression. (b) Schematic depictions of *a*-/*c*-domain orientations based on the activated equivalent slip planes with the maximum *m* = 0.5. (c) Schematic representation of the orientations of the polarization vectors in the deformed sample (black solid box) and the configuration of ^137^Ba nuclear magnetic resonance (NMR) measurements. The rotation axis (X-axis) is perpendicular to the magnetic field B_0_ (Z-axis). An angle of 0° indicates the normal vector of the sample (i.e. [001] direction) as parallel to the magnetic field B_0_. The electric field gradient (EFG) tensor at the barium site for three different domain orientations (*a*_1_, *a*_2_ and *c*) is sensitive to the relative orientation between the EFG tensor and the magnetic field. Therefore, the relative positions of the respective ^137^Ba NMR signal for these domain orientations can be detected by selecting a suitable rotation angle.^[1]^ After the sample was poled along the [110] direction, engineered *a*_1_*-* and *a*_2_*-*domains were obtained from ^137^Ba NMR spectra (referenced to a 1 M BaCl_2_ solution) for selected angles between 0° and 50°, as plotted in (d).


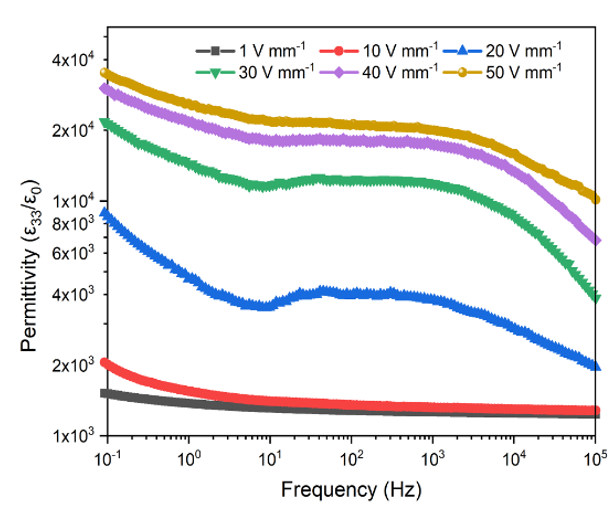


Fig. S15. Frequency-dependent dielectric permittivity for one of the D1150 samples under different fields.





Figure S16. Polarization hysteresis loops obtained at room temperature with a frequency of 1 Hz for various ceramics.


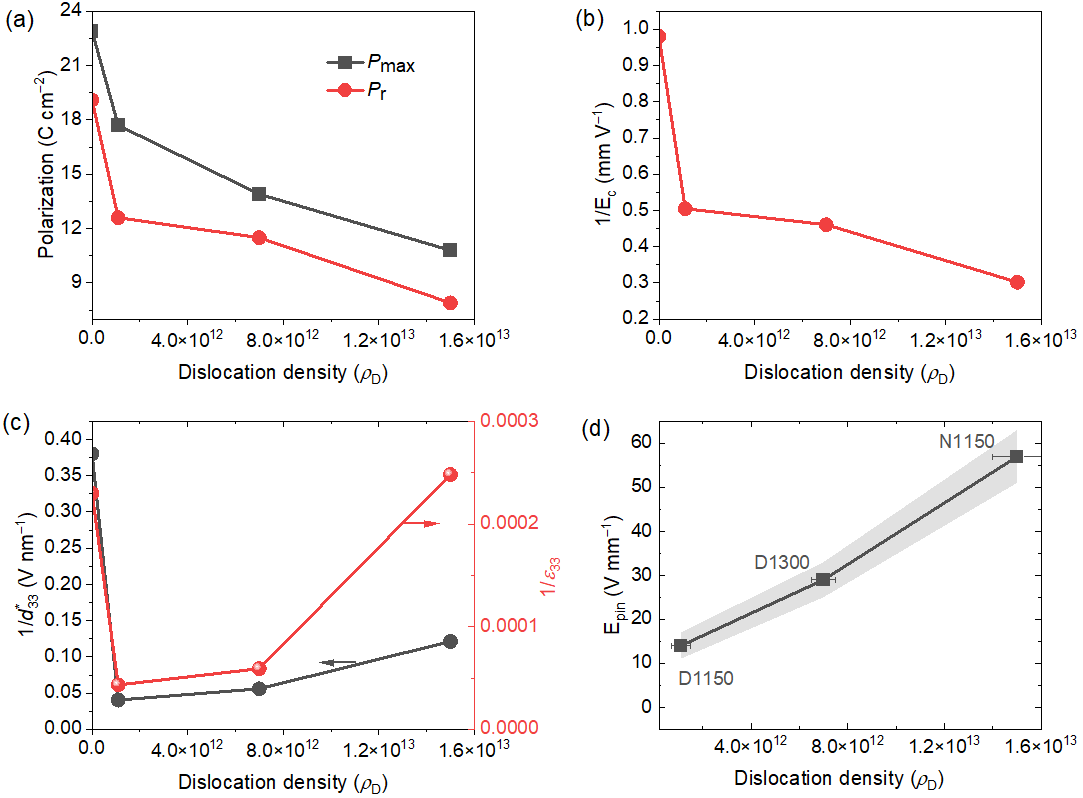


Figure S17. (a) Maximum polarization (*P*_max_) and remanent polarization (*P*_r_), (b) reciprocal of the apparent coercive electric field (*E*_c_), (c) reciprocal of the *d*_33_* and dielectric permittivity (ε _33_), and (d) *E*_pin_ as a function of dislocation density.


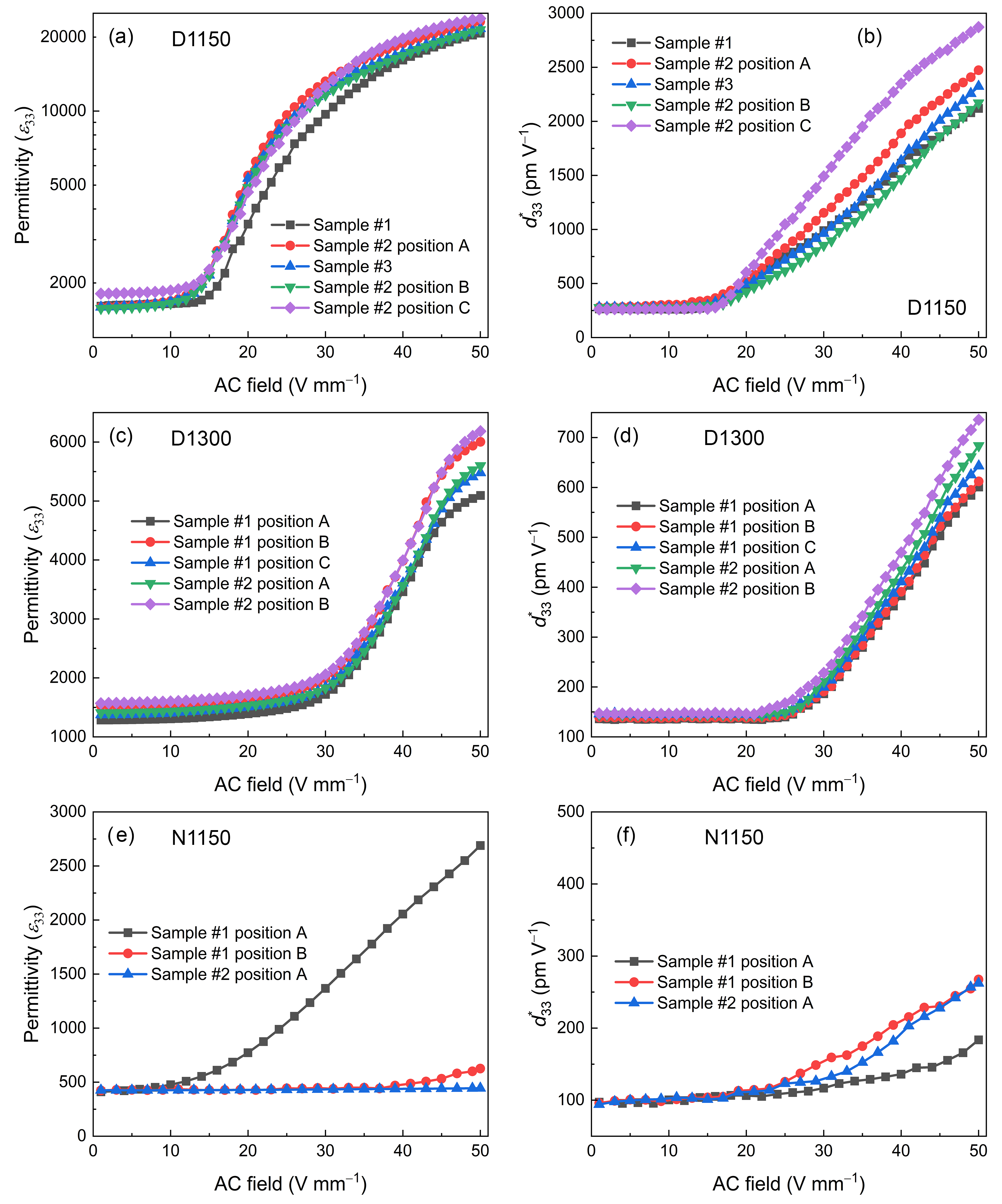


Figure S18. Reproducibility of the engineered relative permittivity and piezoelectric properties. The dislocations are inhomogeneously distributed in the notched samples, therefore permittivity and piezoelectric properties varied largely, as verified in e and f.


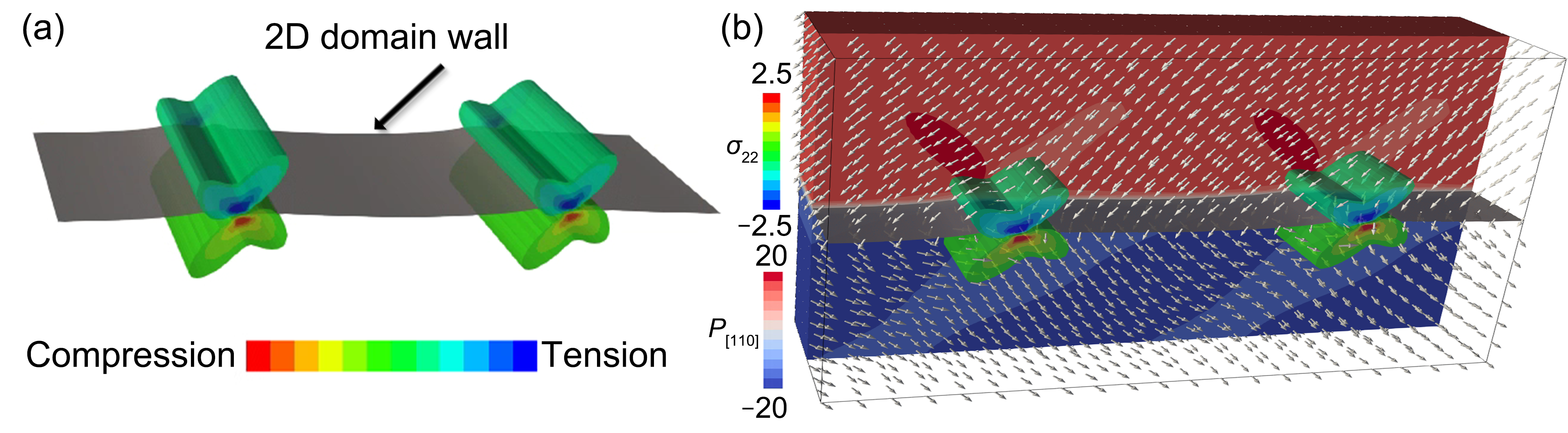


Figure S19. (a) Schematic illustration of two dislocation lines with dislocations parallel to a 2D domain wall. The dislocation-associated stress fields can bend the domain wall and naturally affect the polarization distribution, as exemplified for 90° domain wall-dislocation interactions in (b). The unit for the stress *σ*_22_ and polarization *P*_[110]_ is GPa and μC cm^–2^, respectively.


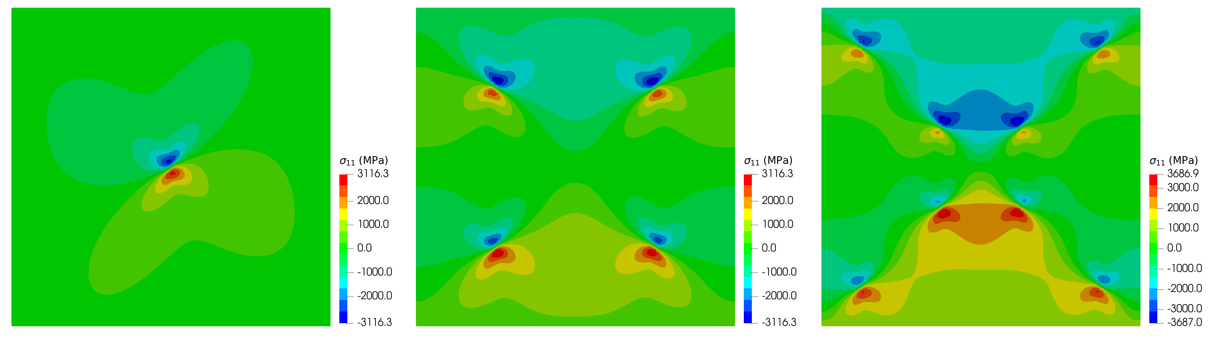


Figure S20. The sets of one, four and eight dislocations used for the simulations of polarization hysteresis loops.


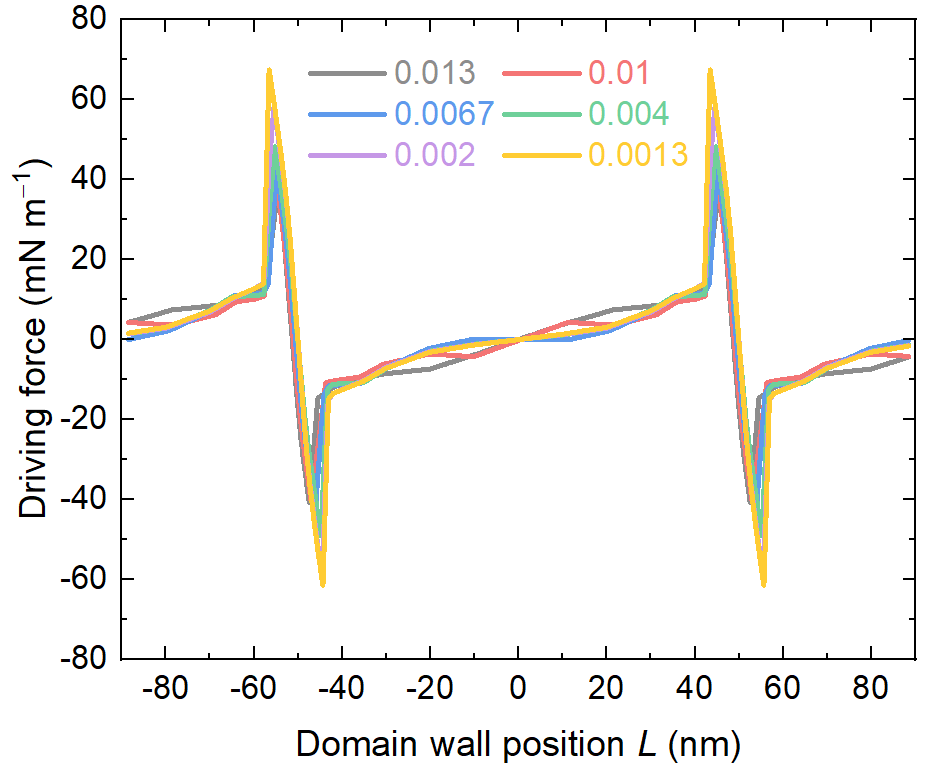


Figure S21. Calculated driving force for domain-wall pinning as a function of domain wall position at different dislocation density. The biggest differences are obtained at the highest driving forces, respectively.


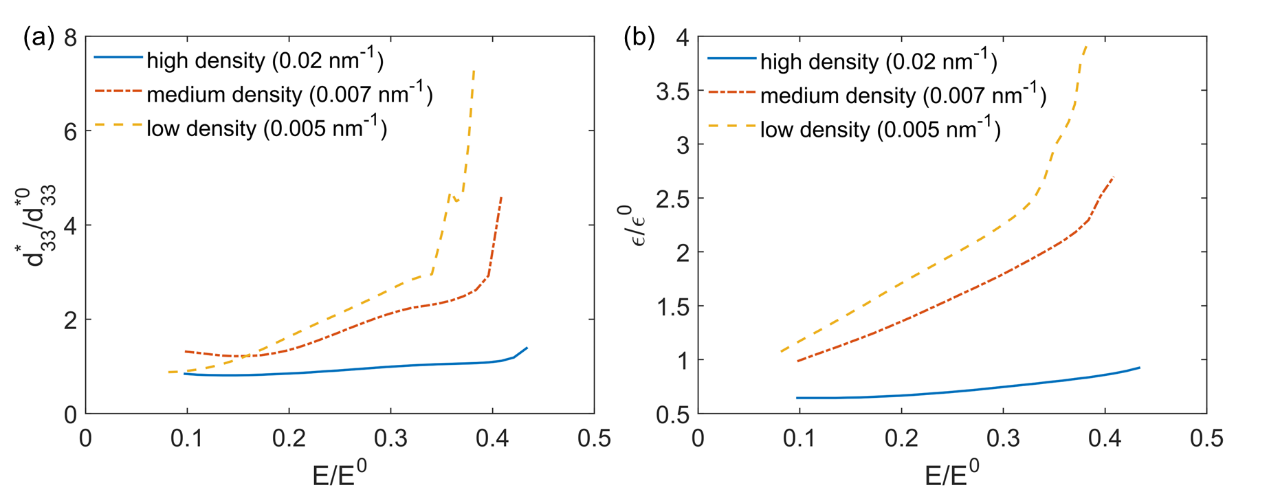


Figure S22. Electric field dependent piezoelectric (*d*_33_) and dielectric (*ε*_33_) properties in (a) and (b) due to dislocations. $d_{33}^{*0}$ = 20 pm V^–1^, *ε*^0^ = 1000 and E_0_ = 9.65 kV mm^–1^.

Table S1. Material parameters of BaTiO_3_ for phase-field simulations.

| Parameters | Symbol | Value | Ref. |
| --- | --- | --- | --- |
| Landau-Devonshire potential coefficients | $\alpha_{1}$ | $4.124\times{10}^{5}(T-115)$ Nm^2^ C^–2^ | [2] |
|  | $\alpha_{11}$ | $-2.097\times{10}^{8}$ Nm^6^ C^–4^ |  |
|  | $\alpha_{12}$ | $7.974\times{10}^{8}$ Nm^6^ C^–4^ |  |
|  | $\alpha_{111}$ | $1.294\times{10}^{9}$ Nm^10^ C^–6^ |  |
|  | $\alpha_{112}$ | $-1.950\times{10}^{9}$ Nm^10^ C^–6^ |  |
|  | $\alpha_{123}$ | $-2.500\times{10}^{9}$ Nm^10^ C^–6^ |  |
|  | $\alpha_{1111}$ | $3.863\times{10}^{10}$ Nm^14^ C^–8^ |  |
|  | $\alpha_{1112}$ | $2.529\times{10}^{10}$ Nm^14^ C^–8^ |  |
|  | $\alpha_{1122}$ | $1.637\times{10}^{10}$ Nm^14^ C^–8^ |  |
|  | $\alpha_{1123}$ | $1.367\times{10}^{10}$ Nm^14^ C^–8^ |  |
| Electrostrictive constants | $Q_{11}$ | $0.1$ C^2^ m^–4^ |  |
|  | $Q_{12}$ | $-0.034$ C^2^ m^–4^ |  |
|  | $Q_{44}$ | $0.029$ C^2^ m^–4^ |  |
| Gradient energy coefficients | $g_{11}$ | $1\times{10}^{-10}$ Vm^3^ C^–1^ | [3] |
| Relative permittivity | $\kappa$ | $200$ | [2] |
| Elastic constants | $c_{11}$ | $178$ GPa |  |
|  | $c_{12}$ | $96.4$ GPa |  |
|  | $c_{44}$ | $122$ GPa |  |
| Dislocation core size | $h$ | $2$ nm |  |

**References**

[1] M. Höfling, X. Zhou, L. M. Riemer, E. Bruder, B. Liu, L. Zhou, P. B. Groszewicz, F. Zhuo, B.-X. Xu, K. Durst, X. Tan, D. Damjanovic, J. Koruza, J. Rödel, *Science* **2021**, *372*, 961.

[2] Y. L. Li, S. Y. Hu, S. Choudhury, M. I. Baskes, A. Saxena, T. Lookman, Q. X. Jia, D. G. Schlom, and L. Q. Chen, *J. Appl. Phys*. **2008**, *104*, 104110.

[3] A. Kontsos and C. M. Landis, *Int. J. Solids Struct.* **2009**, *46*, 1491.
